# A Novel Age-Related Circular RNA Circ-ATXN2 Inhibits Proliferation, Promotes Cell Death and Adipogenesis in Rat Adipose Tissue-Derived Stromal Cells

**DOI:** 10.3389/fgene.2021.761926

**Published:** 2021-11-09

**Authors:** Xing-Hui Song, Ning He, Yue-Ting Xing, Xiao-Qin Jin, Yan-Wei Li, Shuang-Shuang Liu, Zi-Ying Gao, Chun Guo, Jia-Jia Wang, Ying-Ying Huang, Hu Hu, Lin-Lin Wang

**Affiliations:** ^1^ Core Facilities, Zhejiang University School of Medicine, Hangzhou, China; ^2^ Department of Basic Medicine Sciences and Department of Orthopaedics of Sir Run Run Shaw Hospital, Zhejiang University School of Medicine, Hangzhou, China; ^3^ China Medical Research Center, Zhejiang Chinese Medical University Academy of Chinese Medical Sciences, Hangzhou, China; ^4^ School of Medicine, Zhejiang University City College, Hangzhou, China; ^5^ Department of Pathology and Pathophysiology, Zhejiang University School of Medicine, Hangzhou, China

**Keywords:** aging, circ-ATXN2, proliferation, adipogenesis, cell death, ASCs

## Abstract

Adipose tissue-derived stromal cells are promising candidates investigating the stem cell-related treatment. However, their proportion and utility in the human body decline with time, rendering stem cells incompetent to complete repair processes *in vivo*. The involvement of circRNAs in the aging process is poorly understood. Rat subcutaneous adipose tissue from 10-week-old and 27-month-old rats were used for hematoxylin and eosin (H and E) staining, TUNEL staining, and circRNA sequencing. Rat adipose tissue-derived stromal cells were cultured and overexpressed with circ-ATXN2. Proliferation was examined using xCELLigence real-time cell analysis, EdU staining, and cell cycle assay. Apoptosis was induced by CoCl2 and examined using flow cytometry. RT-PCR assay and Oil Red O staining were used to measure adipogenesis at 48 h and 14 days, respectively. H and E staining showed that the diameter of adipocytes increased; however, the number of cells decreased in old rats. TUNEL staining showed that the proportion of apoptotic cells was increased in old rats. A total of 4,860 and 4,952 circRNAs was detected in young and old rats, respectively. Among them, 67 circRNAs exhibited divergent expression between the two groups (fold change ≥2, *p* ≤ 0.05), of which 33 were upregulated (49.3%) and 34 were downregulated (50.7%). The proliferation of circ-ATXN2-overexpressing cells decreased significantly *in vitro*, which was further validated by xCELLigence real-time cell analysis, EdU staining, and cell cycle assay. Overexpression of circ-ATXN2 significantly increased the total apoptotic rate from 5.78 ± 0.46% to 11.97 ± 1.61%, early apoptotic rate from 1.76 ± 0.22% to 5.50 ± 0.66%, and late apoptosis rate from 4.02 ± 0.25% to 6.47 ± 1.06% in adipose tissue-derived stromal cells. Furthermore, in circ-ATXN2-overexpressing cells, RT-PCR assay revealed that the expression levels of adipose differentiation-related genes *PPARγ* and *CEBP/α* were increased and the Oil Red O staining assay showed more lipid droplets. Our study revealed the expression profile of circRNAs in the adipose tissue of old rats. We found a novel age-related circular RNA—circ-ATXN2—that inhibits proliferation and promotes cell death and adipogenesis in rat adipose tissue-derived stromal cells.

## Introduction

Adipose tissue consists of multiple cell types. The proportion and function of these cells vary across different life stages (Stout et al., 2017). Adipose tissue-derived stromal cells (ASCs) are rich in adipose tissue and also called adipose tissue-derived mesenchymal stem cells. ASCs possess the potential of multilineage differentiation and immunoregulatory properties. They are used as a model material for stem cell-based therapies (Fraser et al., 2006; Frese et al., 2016), such as anti-aging or neurorestoration (Huang et al., 2020). However, their proportion and utility in the human body decline with time, making stem cells incompetent to complete repair processes *in vivo* (H. Wang 1989; Liu et al., 2014; Zhang et al., 2020) and stem cell-based therapies. It was determined that the cell proliferation, apoptosis, and differentiation potential of ASCs were different from mice at different ages and suggested that the donor’s age should be cautiously considered when applying ASCs to tissue-specific cell-based regenerative therapies (Zhang et al., 2020).

Stem cells can be used for tissue regeneration (Cai et al., 2021). Previous studies have reported that the repair capacity of stem cells in older individuals may be improved by genetically reprogramming stem cells to exhibit delayed senescence and possess enhanced regenerative properties (Madonna et al., 2013; Chao Chen 2020; Xiang et al., 2020). Non-coding RNAs are emerging regulators in physiological processes (Sun et al., 2013; Kim et al., 2014; Arner and Kulyte 2015; Knoll et al., 2015). Among them, circRNAs feature covalently closed loops without 5′ or 3′ polarity and are classified as new endogenous non-coding RNAs. Recently, numerous studies have reported the functions of circRNAs in cell proliferation and apoptosis and adipogenesis (Davidson et al., 2012; Arcinas et al., 2019; Liu et al., 2020; Meng et al., 2020; Zhu et al., 2020). Moreover, studies have indicated that circRNAs function as important regulators of ASCs adipogenesis in the adipose tissue (Zhu et al., 2020). However, the functions of age-related circRNAs in ASCs are poorly understood.

In this study, to identify the age-related circRNA functions in ASCs, we sequenced circRNA from rat subcutaneous adipose tissue (SAT) in the 10-week-old and 27-month-old rats. This study reveals the profiles of circRNAs in the SAT of aging rats for the first time. The data showed that the changes in circRNA expression were specific to aging. Moreover, we discovered a correlation between age-related circRNA and mRNA expression in the host. To further investigate the regulation of age-related circRNA in ASCs, we overexpressed circ-ATXN2 in rat ASCs. We found that circ-ATXN2 overexpression inhibited proliferation and promoted cell death and adipogenesis in ASCs. Our results highlight the role of circRNAs in the maintenance of ASCs function during aging and suggest that circRNAs are potential targets in ASCs in cell-based regenerative and anti-aging therapies.

## Materials and Methods

### Animals and Sample Collection

The animal study was reviewed and approved by the Animal Ethics Committee of Zhejiang University (ZJU 2015-169-01). Male Sprague Dawley rats in the young (10 weeks, n = 3) and old groups (27 months, n = 3) were provided by the Animal Center of Zhejiang University (Zhejiang, China). The rats were housed in a room with constant temperature (20–25°C), humidity (50–65%), and light (12-h light/12-h dark cycle) control and had free access to food and water. SATs were collected from all the rats that were sacrificed using CO_2_ asphyxiation. A part of the SAT was immediately frozen in liquid nitrogen and stored at −80°C for subsequent analysis and RT-PCR. In addition, some parts of the SAT were fixed using 4% formaldehyde for hematoxylin and eosin (H and E) and TUNEL staining.

### Adipose Tissue Characteristic Analysis

The diameter and number of adipocytes were determined in 3-4 paraffin-embedded sections of the SAT. The sections were stained with H and E. After staining, the diameters of adipocytes were determined in three sections from young (n = 100 adipocytes) and old rats (n = 100 adipocytes), using the NDP. VIEW image software of NanoZoomer S60(HAMAMATSU, Japan). The number of adipocytes in the same area was counted from three sections from the two groups of rats (n = 3) using the NDP. VIEW image software.

The TUNEL assay was performed in each group (n = 3) using the Apoptotic Detection kit (Cat# MK500, Takara, Japan). All stained sections were imaged under a confocal microscope (Nikon, Japan).

The immunofluorescence assay was performed as previously described (Jeong et al., 2020). Briefly, sections (n = 3) were incubated in primary antibodies Cleaved Caspase-3 (Cat#9664, CST, United States) at 4 °C overnight and with a secondary antibody for 1 h at room temperature. All stained sections were imaged under a confocal microscope (Nikon, Japan).

### RNA Sequencing and Functional Analysis

Total RNA was isolated from the SATs of young and old rats using the Trizol reagent (Invitrogen, United States). Approximately 5 µg of total RNA was used to deplete ribosomal RNA according to the manuscript of the Ribo-Zero™ rRNA Removal Kit (Illumina, San Diego, United States). After removing ribosomal RNAs, total RNA samples were qualified and purified. The Illumina Hiseq 4,000 (LC Bio, China) was used to screen the circRNA expression profile of 9,812 circRNAs. Raw RNA-seq data were deposited in the Gene Expression Omnibus database (https://www.ncbi.nlm.nih.gov; accession number: SUB10360317). Thereafter, we used FastQC (http://www.bioinformatics.babraham.ac.uk/projects/fastqc/) to verify the sequence. The differentially expressed circRNAs between the two groups were selected with a fold change ≥2 and statistical significance (*p* value ≤0.05) using the R package “edgeR” (Robinson et al., 2010). Using the “clusterProfiler” package in the R studio, Gene Ontology (GO) and Kyoto Encyclopedia of Genes and Genomes (KEGG) enrichment analyses were performed to explore the potential role of circRNAs. To reveal the functions and mechanisms of the circRNAs, TargetScan and miRanda were used to predict miRNA–circRNA interactions.

### Cell Culture and Treatment

Male Sprague Dawley rats (4-day-old) were provided by the Animal Center of Zhejiang University. ASCs were isolated from their SAT and cultured as previously described (Song et al., 2017). Briefly, the SAT was digested adequately with 1% collagenase (Gibco, United States) and centrifuged at 800 × *g* for 3 min. Thereafter, cells were cultured in a culture medium containing low-sugar Dulbecco’s modified Eagle medium (DMEM; Gibco), 10% fetal bovine serum (FBS; Gibco), and penicillin (100 U/ml)/streptomycin (100 μg/ml). The culture medium was changed every 3 days. Our previous flow cytometry analysis showed that the isolated ASCs had high purity based on positivity for CD90 and CD105 (Song et al., 2017).

### Lentivirus Construction and Infection

To overexpress circ-ATXN2, we used the pLC5-ciR-GFP (#GS0108) vector (Geneseed, Guangzhou, China). We inserted the full-length circ-ATXN2 (chr12: 40329454-40335680) into the pLC5-ciR-GFP vector between the restriction sites. Thereafter, the vector was packaged into the lentivirus and infected into rat ASCs (Sun et al., 2020). The vector-GFP- and circ-ATXN2–GFP-expressing cells were sorted for subsequent experiments using a Moflo Astrios^EQ^ flow cytometer (Beckman Coulter, United States).

### Cell Proliferation Assay

#### xCELLigence Real-Time Cell Analysis (RTCA) System Assay for Proliferation

According to previous reports (Stoddart 2011; Fabio Cerignoli et al., 2018), the RTCA system (ACEA Biosciences, DP) continuously measured cell viability in real-time through the impedance readout. The arbitrary unit reflecting the electronic cell-sensor impedance is called the cell index (CI). We added 50 μl of ASC culture medium to the wells of 16-E-Plates (ACEA Biosciences; n = 4) and measured the background impedance. The vector-GFP- and circ-ATXN2–GFP-expressing cells were seeded at a density of 2,000 cells per well of a 16-E-Plate in 100 μl of culture medium_ and allowed to passively adhere to the electrode surface. The 16-E-Plate was placed inside a laminar flow hood for half an hour at 24–26°C before being transferred into a cell culture incubator with an RTCA instrument. Data were recorded at 15-min intervals throughout the entire experiment once the plates were properly placed.

#### 5-Ethyny1-2′-denoxyridine (EdU) Incorporation Assay for Proliferation

An EdU proliferation kit (iFluor647, Abcam) was used to detect cell proliferation. Following the protocols described earlier (Liu et al., 2019), we seeded vector-GFP- and circ-ATXN2–GFP-expressing cells (1 × 10^4^) onto 96-well plates (Corning) for 24 h. The cells were treated with EdU solution (50 µM) for 4 h at 37°C. Thereafter, the cells were fixed with 4% paraformaldehyde. Cells were then permeabilized with PBS supplemented with 0.5% Triton X-100 for 10 min and stained with Hoechst 33,342 for 30 min in the dark. Finally, the cells were viewed under a confocal microscope (IX81-FV1000, Olympus, Japan).

For flow cytometry, vector-GFP- and circ-ATXN2–GFP-expressing cells (5 × 10^5^) were seeded into 6-well plates for 24 h. Following the above methods, a flow cytometer (Cytoflex LX, Beckman Coulter, United States) was employed to detect the percentage of EdU-positive cells.

### Flow Cytometry Assay for Cell Cycle

Cell cycle assays were conducted as previously described (Hall et al., 2019). Briefly, vector-GFP- and circ-ATXN2–GFP-expressing cells (1 × 10^6^) were collected and washed with PBS. Next, 70% ethanol was used to fix the cells, and they were kept at 4°C overnight. The cells were dyed with 3.0 μg/ml propidium iodide (PI, Sigma) supplemented with 12.5 μg/ml RNase (Gibco, United States). An FC500 flow cytometry system (Beckman Coulter) was used to measure the number of cells in the different phases of the cell cycle.

### Flow Cytometry Assay for Apoptosis

To observe the effects of circ-ATXN2 overexpression on cell death, CoCl_2_ was used to create apoptotic models of the cells. Vector-GFP- and circ-ATXN2–GFP-expressing cells were seeded at 1 × 10^5^ cells per well in 6-well plates and incubated with 500 μM CoCl_2_ for 24 h. Annexin V-APC/PI apoptosis kit (MULTI SCIENCES) was used to determine cell apoptosis. Cells were collected in 500 μl of binding buffer with 3 μl Annexin V-APC and 3 μl PI for 20 min at room temperature in the dark. Apoptotic cells were detected using a flow cytometer (DxFLEX, Beckman Coulter, United States) (He et al., 2021; Song et al., 2021).

### Adiopogenesis Assay

When cell confluence reached 100%, DMEM containing 10% FBS, 1 μmol/L dexamethasone, 0.5 mmol/ml 3-isobutyl-1-1-methylxanthine, and 5 μg/L insulin (Sigma, Shanghai, China) were used to induce adipogenesis in vector-GFP- and circ-ATNX2–GFP-expressing cells. The medium was changed every 3 days. The genes associated with adipogenesis were detected using RT-PCR 48 h after induction. We performed Oil Red O staining 14 days after adipogenesis induction. The differentiated adipocytes were imaged using a microscope (Zeiss, Germany).

### RT-PCR Assay

Total RNA from rat the SATs of young and old rats was extracted using the Trizol reagent (Invitrogen) as described previously. RNA was treated with RNase R (3 U/μg, Geneseed) for 30 min. The expression levels of circ-ATNX2 were measured using RT-PCR. To detect the expression of genes associated with adipogenic differentiation, the total RNA of vector-GFP and circ-ATNX2-GFP-expressing cells was extracted using the Trizol reagent (Invitrogen). All reactions were performed using SYBR green kits on 480 II systems (Roche, United States) thrice. The expression of all genes was normalized to that of GAPDH. The primer sequences used for RT-PCR in this study are listed in Table 1. Experiments were repeated three times independently.

**TABLE 1:** RT-PCR primer sequences.

Genes	Forward (5′-3′)	Reverse (5′-3′)
circ-ATXN2	AGT​TAT​GCA​CGA​AGA​GCC​ACC​T	AGA​AAT​CGT​AGG​CTG​AGG​CAG
PPARγ	ACC​ACA​GTT​GAT​TTC​TCC​AG	TGT​TGT​AGA​GCT​GGG​TCT​TT
CEBP/α	CGA​CTT​CTA​CGA​GGT​GGA​G	ATG​TAG​GCG​CTG​ATG​TCT​AT
GAPDH	CCA​CCA​CCC​TGT​TGC​TGT​AG	CTT​GGG​CTA​CAC​TGA​GGA​CC

### Oil Red O Staining and Quantification

To detect the production of lipid droplets, the cell medium was removed, and then, the cells were fixed with 4% formaldehyde at room temperature. To stain the lipid droplets in cells, we treated the cells with filtered Oil Red O solution for 20 min at room temperature and then removed the residual dyes with 60% isopropanol. Oil Red O dyes within cells were extracted with 100% isopropanol, and the absorbance was measured at 540 nm to quantify the lipid droplets within the cells (Zhang et al., 2019).

### Statistical Analyses

All experiments were performed at least thrice. GraphPad Prism 8.0 was used to graphically represent the results and conduct statistical analyses. Results are presented as the mean ± SEM. A *p* value <0.05 was considered statistically significant using the Student’s *t*-test or two-way ANOVA.

## Results

### Increased Size and Cell Death of Adipocytes in Aged Rat Adipose Tissue

**FIGURE 1:**
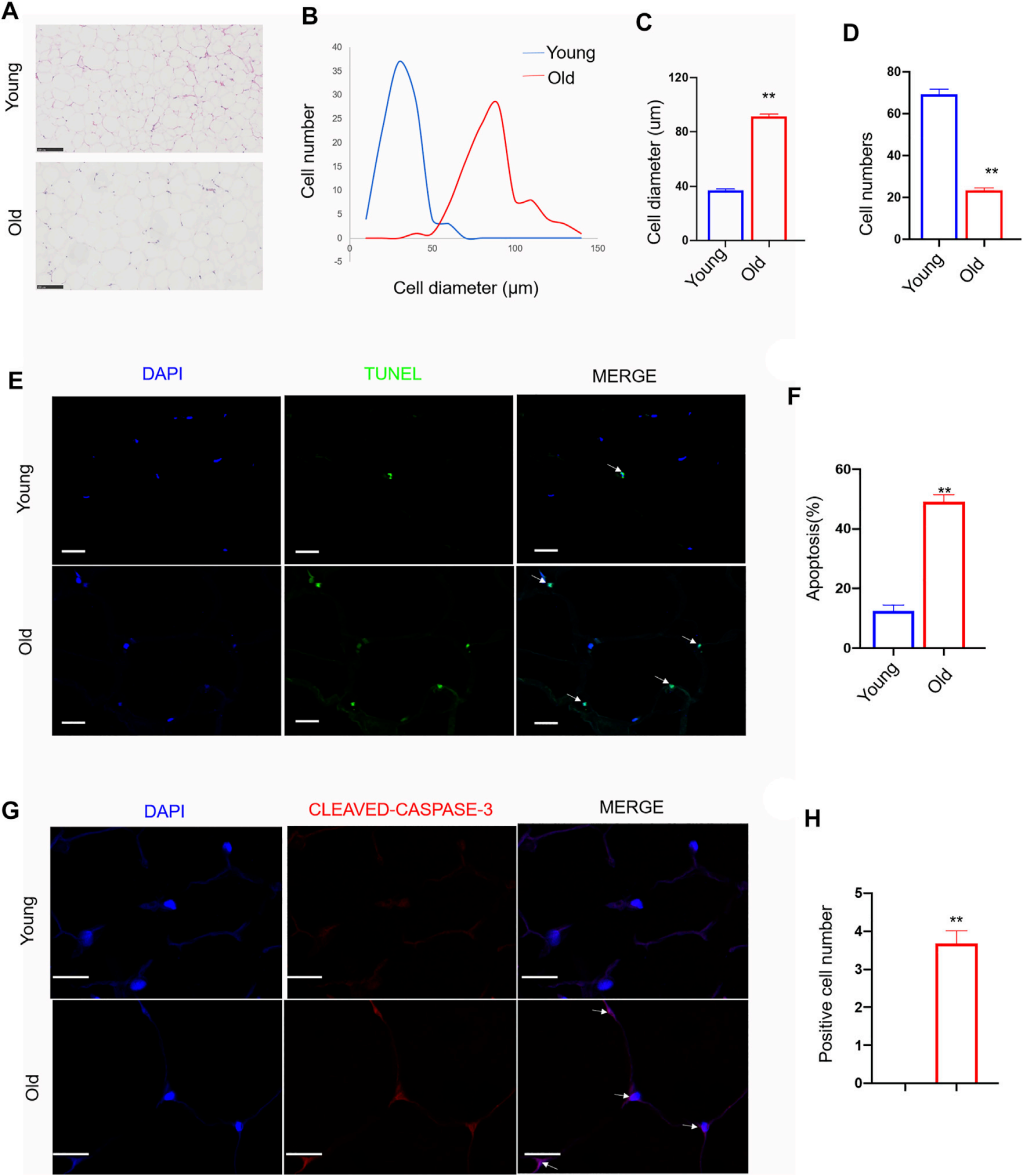
Effect of aging on cell morphology, diameter, number and apoptosis in rat subcutaneous adipose tissue. Subcutaneous adipose tissue was isolated from young and old rats. **(A)** Representative images (200x) of Hematoxylin and eosin staining (H and E) in adipose tissue sections from young group and old group rat. scale bars: 100 µm. **(B–D)**. Quantification of diameter and number of adipocytes in section. **(E)** Representative immunofluorescence staining of TUNEL (green) and DAPI (blue) of the adipose tissue sections (n = 3). Scale bars: 20 µm. **(F)** Quantification of apoptosis rate in adipose tissue sections. **(G)** Representative immunofluorescence staining of cleaved-caspase-3 (red) and DAPI (blue) of the adipose tissue sections (n = 3). Scale bars: 20 µm. **(H)** Quantification of positive cell number of cleaved-caspase-3 in sections. The data are represented as mean ± SEM (n = 100 cells per group). **p < 0.01, *vs* young group (Student’s *t* test).

As shown in Figures 1A–C, the size of adipocytes was significantly increased in old rats. The average cell diameter of young rats was 36.85 ± 9.95 µm, whereas the average cell diameter of old rats was 91.30 ± 17.69 µm (*p* < 0.01). The number of adipocytes in the same size area was significantly lower in old rats (Figure 1D). The number of adipocytes in young rats was 69.2 ± 7.74, whereas the number of adipocytes in old rats was 23.4 ± 3.23. (*p* < 0.01). Next, TUNEL assay showed that the number of TUNEL staining positive adipocytes was increased in old group (Figures 1E,F). It was 12.43 ± 4.12% in young rats, whereas it was 49.18 ± 4.69% in old group (*p* < 0.01). Immunofluorescence assay showed the similar apoptosis trend in old group (Figures 1G,H). The expression level of cleaved-caspase-3 was significantly increased in old group (*p* < 0.01).

### RNA-Seq Data Profile

**FIGURE 2:**
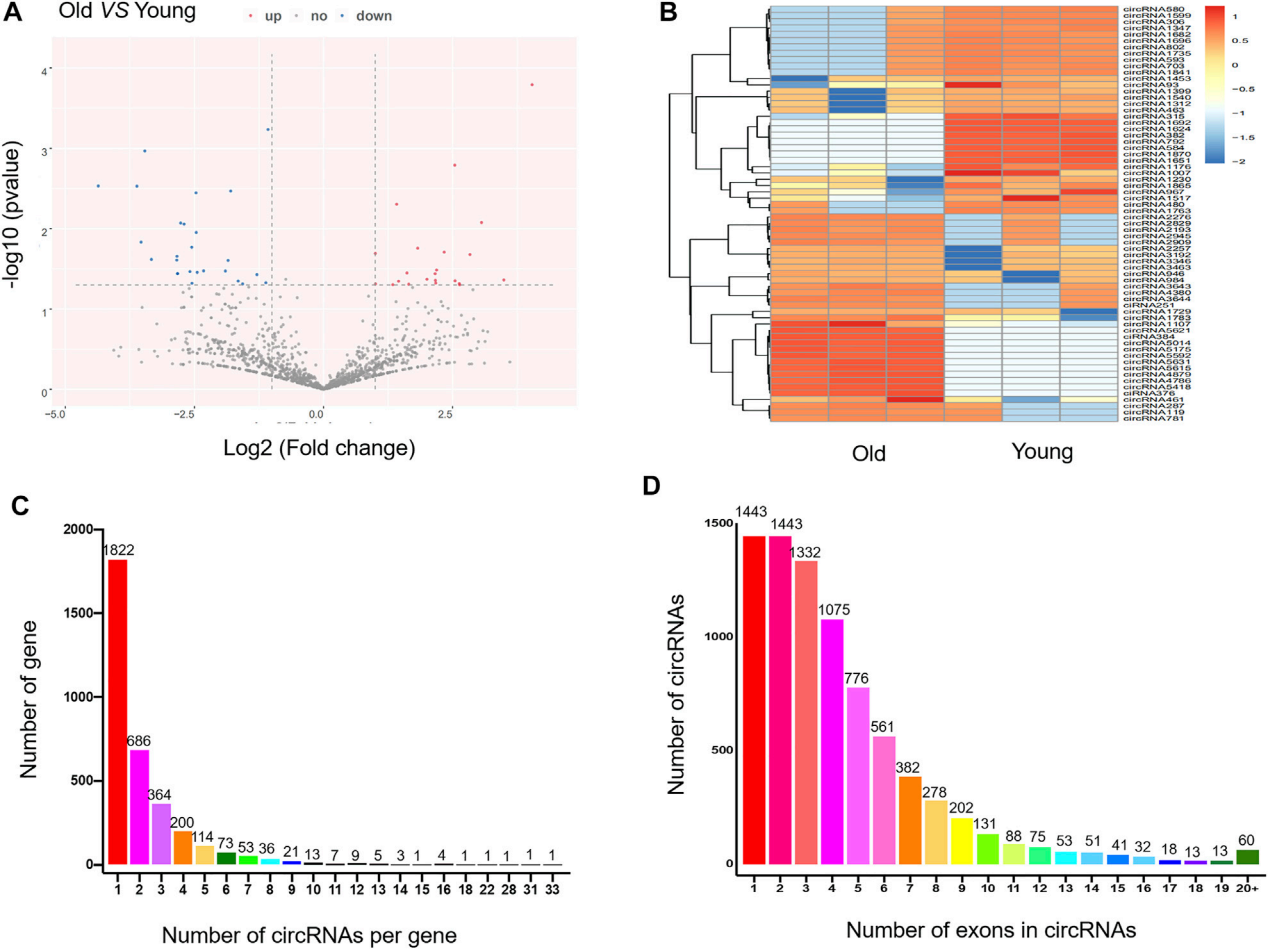
Expression profile of circRNAs in adipose tissue in young and old rats. **(A)** The volcano plot for the differentially expressed circRNAs. In the volcano plot, the *X*-axis represents fold-change (log2) and *Y*-axis represents P (-*log*10). Red points (fold change ≥2) indicate up-regulated circRNAs, blue points (fold change ≤2) indicate down-regulated circRNAs. **(B)** The heatmap for the differentially expressed circRNAs. In clustering analysis, up- and down-regulated genes are colored in red and blue, respectively. **(C)** The number of circRNAs per gene. **(D)** The number of exons in circRNAs.

Ribosomal RNA-depleted total RNA sequencing (RNA-seq) analysis was used to map the circRNAs in the rat SAT during aging. We used CIRI and CIRCExplorer2 software to predict circRNAs. According to the identification criteria, including mismatch ≤2, back-spliced junction reads ≥1, and two splice site distances over the genome were less than 100 kb, 4,860 and 4,952 circRNAs were detected in young rats and old rats, respectively. Among them, 67 circRNAs were differentially expressed (DEcircRNAs) (fold change ≥2, *p* ≤ 0.05) between the two groups, of which 33 were upregulated (49.3%) and 34 were downregulated (50.7%). The expression features of DEcircRNAs are depicted using a volcano plot (Figure 2A) and two-dimensional hierarchical clustering heatmap (Figure 2B). The top 20 DEcircRNAs are listed in Table 2. In addition, there are 8,067 circRNAs with exons, and the number of exons within circRNAs ranged from 1 to more than 20; however, it was most common for them to harbor less than 10 exons. Only 5.5% contained 11 exons (Figure 2C). Furthermore, 1,596 of the 3,418 genes generated two or more circRNAs (Figure 2D).

**TABLE 2:** Top 20 of differential expressed circRNAs between young group and old group in rat subcutaneous adipose tissue.

circRNA	log2FoldChange	Regulation	chr	Strand	geneName	P value
circRNA2276	4.04	up	chr19	+	Arhgap10	0
circRNA315	−1.07	down	chr17	+	Fam120a	0
circRNA1599	−3.46	down	chr4	+	ENSRNOG00000023679	0
circRNA2257	2.54	up	chr14	-	Sptbn1	0
circRNA580	−4.36	down	chr18	-	Smad4	0
circRNA306	−3.61	down	chr17	-	Naa35	0
circRNA1176	−1.8	down	chr2	+	RGD1307100	0
circRNA1230	−2.47	down	chr2	-	Tet2	0
circRNA1107	1.42	up	chr3	-	Ralgapa2	0
circRNA287	3.06	up	chr10	+	Tlk2	0.01
circRNA1312	−2.77	down	chr1	+	C2cd3	0.01
circRNA480	−2.7	down	chr14	-	Nf2	0.01
circRNA1729	1.82	up	chr6	-	intergenic_circRNA	0.02
circRNA2945	2.34	up	chr1	-	Cpeb3	0.02
circRNA1783	1	up	chr12	-	ENSRNOG00000001256	0.02
circRNA3644	2.84	up	chr16	+	Capn7	0.02
circRNA802	−2.84	down	chr5	+	Coq3	0.02
circRNA593	−3.33	down	chr20	+	Bag6	0.02
circRNA119	2.19	up	chr12	-	Atp6v0a2	0.03
circRNA3192	1.62	up	chr10	-	intergenic_circRNA	0.04

### DEcircRNAs Function and circRNA–miRNA Analysis

**FIGURE 3:**
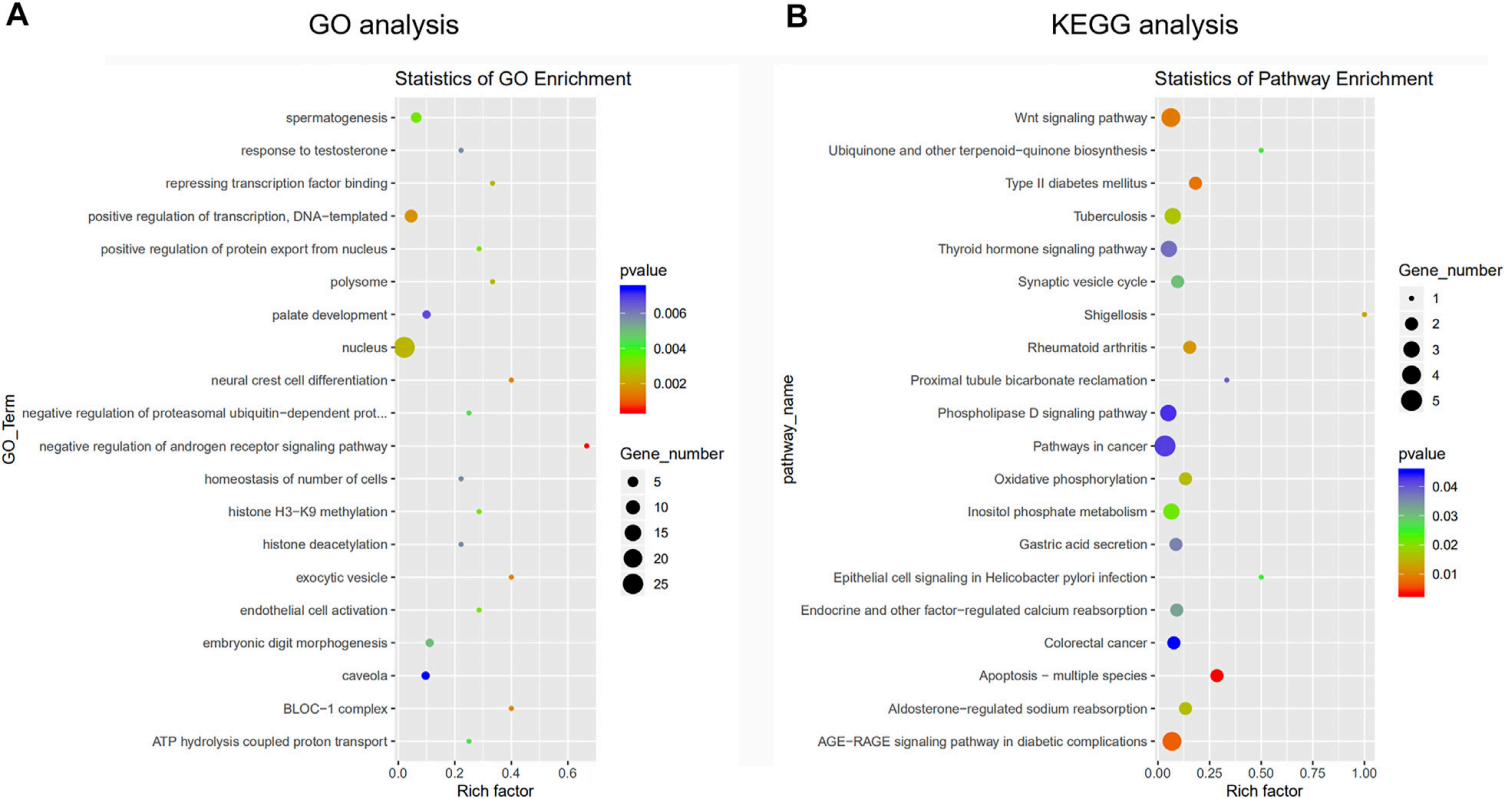
Gene ontology (GO) analysis and Kyoto Encyclopedia of Genes and Genomes (KEGG) pathway annotation of differentially expressed circRNAs between young group and old group. **(A)** The bubble chart shows the top 20 most significantly enriched and meaningful GO terms related to the altered circRNAs ranged according to the predicted *p*-values. **(B)** The bubble chart shows the top 20 most significantly enriched pathways depending on age. The enrichment factor on the *X*-axis indicates the specificity of a pathway activating in old samples compared with young samples. The bubble size represents the number of circRNAs enriched in a GO terms or pathway. The red bubble indicates a more significant GO terms or pathway.

GO and KEGG analyses were performed to understand the main potential function and mechanisms of DEcircRNAs in the rat SATs between the young and old groups based on their host genes. The most enriched GO items and KEGG pathways are shown in the bubble charts (Figure 3). The GO terms of the DEcircRNAs related to aging are shown in Table 3. GO analysis indicated that the host genes of DEcircRNAs in aging were mostly involved in apoptosis (such as GO1900740, GO2000055, GO0090244, GO0007258, GO0071936, GO0004705, GO0070412, and GO0046332), proliferation (GO0042127, GO0048872, GO0002053, and GO0060284), and differentiation (GO0045655). In addition, KEGG analysis revealed that the host genes of DEcircRNAs were mainly concentrated in aging-related pathways, including the Wnt signaling pathway, type II diabetes mellitus, rheumatoid arthritis, apoptosis-multiple species, and AGE–RAGE signaling pathway in diabetic complications. The KEGG analysis of DEcircRNAs related to adipose tissue aging (apoptosis, proliferation, and differentiation) are shown in Table 4. TargetScan and miRanda software were used to analyze the functions and mechanisms of the circRNAs, and they showed that 97% (65/67) of the DEcircRNAs shared miRNA binding sites.

**TABLE 3:** GO terms of DEcircRNAs related to adipose tissue aging (apoptosis, proliferation and differentiation).

GO_ID	GO_Term	GO_function	Function	P value
GO:0045893	positive regulation of transcription, DNA-templated	biological_process	apoptosis	0
GO:0048872	homeostasis of number of cells	biological_process	proliferation	0.01
GO:0042127	regulation of cell proliferation	biological_process	proliferation	0.01
GO:0002053	positive regulation of mesenchymal cell proliferation	biological_process	proliferation	0.01
GO:1900740	positive regulation of protein insertion into mitochondrial membrane involved in apoptotic signaling pathway	biological_process	Apoptosis	0.01
GO:2000055	positive regulation of Wnt signaling pathway involved in dorsal/ventral axis specification	biological_process	proliferation/apoptosis/differentiation	0.01
GO:2001182	regulation of interleukin-12 secretion	biological_process	Apoptosis	0.01
GO:0090244	Wnt signaling pathway involved in somitogenesis	biological_process	proliferation/differentiation	0.01
GO:0072619	interleukin-21 secretion	biological_process	Apoptosis	0.01
GO:0016241	regulation of macroautophagy	biological_process	Apoptosis	0.01
GO:0050706	regulation of interleukin-1 beta secretion	biological_process	Apoptosis	0.01
GO:0060284	regulation of cell development	biological_process	proliferation	0.01
GO:0045655	regulation of monocyte differentiation	biological_process	differentiation	0.01
GO:0007258	JUN phosphorylation	biological_process	Apoptosis	0.01
GO:1901256	regulation of macrophage colony-stimulating factor production	biological_process	Apoptosis	0.01
GO:0030511	positive regulation of transforming growth factor beta receptor signaling pathway	biological_process	proliferation/apoptosis/differentiation	0.01
GO:0046332	SMAD binding	molecular_function	apoptosis	0.01
GO:0071936	coreceptor activity involved in Wnt signaling pathway	molecular_function	proliferation/apoptosis/differentiation	0.01
GO:0005009	insulin-activated receptor activity	molecular_function	apoptosis	0.01
GO:0004705	JUN kinase activity	molecular_function	apoptosis	0.01
GO:0070412	R-SMAD binding	molecular_function	apoptosis	0.01

**TABLE 4:** KEGG enrichment of DEcircRNAs related to adipose tissue aging (apoptosis, proliferation and differentiation).

pathway_id	pathway_name	circRNA (host genes)	Function
ko04215	Apoptosis - multiple species	circRNA2909(Htra1); circRNA4786 (Mapk8)	apoptosis/proliferation/differentiation
ko04933	AGE-RAGE signaling pathway in diabetic complications	circRNA1624 (ENSRNOG00000003357); circRNA4786 (Mapk8); circRNA580(Smad4); ciRNA376 (ENSRNOG00000033119)	apoptosis/proliferation/differentiation
ko04930	Type II diabetes mellitus	circRNA4786 (Mapk8); circRNA93(Insr)	apoptosis/proliferation/differentiation
ko04310	Wnt signaling pathway	circRNA1007(Lrp6); circRNA4786 (Mapk8); circRNA580(Smad4); ciRNA376 (ENSRNOG00000033119)	apoptosis/proliferation/differentiation
ko04960	Aldosterone-regulated sodium reabsorption	circRNA1540(Atp1b3); circRNA93(Insr)	apoptosis/proliferation
ko00190	Oxidative phosphorylation	circRNA119(Atp6v0a2); circRNA792(Atp6v1h)	apoptosis/proliferation/differentiation
ko00562	Inositol phosphate metabolism	circRNA4879(Inpp4b); circRNA5418(Pi4k2a); ciRNA376 (ENSRNOG00000033119)	apoptosis/proliferation/differentiation
ko04961	Endocrine and other factor-regulated calcium reabsorption	circRNA1540(Atp1b3); ciRNA376 (ENSRNOG00000033119)	apoptosis/proliferation
ko05200	Pathways in cancer	circRNA3643(Arhgap21); circRNA4786 (Mapk8); circRNA580(Smad4); circRNA584(Pias2); ciRNA376 (ENSRNOG00000033119)	apoptosis/proliferation/differentiation
ko04072	Phospholipase D signaling pathway	circRNA1107(Ralgapa2); circRNA93(Insr); ciRNA376 (ENSRNOG00000033119)	apoptosis/proliferation
ko05210	Colorectal cancer	circRNA4786 (Mapk8); circRNA580(Smad4)	apoptosis/proliferation/differentiation
ko04214	Apoptosis - fly	circRNA1865 (ENSRNOG00000010815)	apoptosis
ko04974	Protein digestion and absorption	circRNA1540(Atp1b3); circRNA1624 (ENSRNOG00000003357)	apoptosis/proliferation
ko04911	Insulin secretion	circRNA1540(Atp1b3); ciRNA376 (ENSRNOG00000033119)	apoptosis/proliferation

### Characterization of Circ-ATXN2 in Rat SAT

**FIGURE 4:**
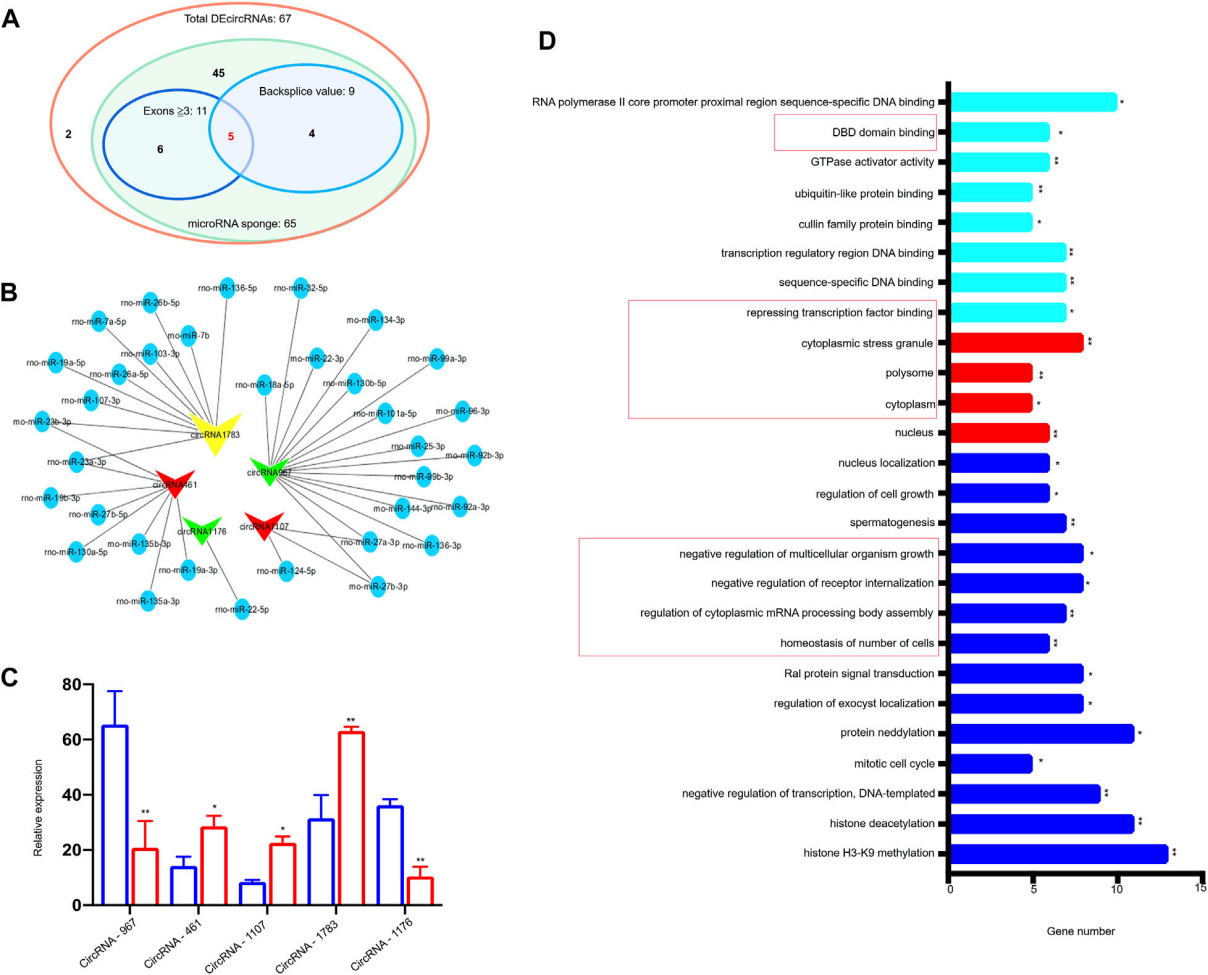
Characterization of 5 DEcircRNAs in rat subcutaneous adipose tissue. **(A)** Venn show abundant (exons ≥3) exonic DEcircRNAs with potential miRNA binding sites and DEcircRNAs with backsplice value of every sample. **(B)** Bioinformatics prediction of 5 circRNAs, their predicted miRNAs, and miRNA targets. circRNAs were plotted as V-shapes. Yellow V-shapes represent circRNA-1783 (circ-ATXN2), which is an up-regulated circRNA. Green V-shapes represent down-regulated circRNAs (circRNA-967, circRNA-1176). Red V-shapes represent up-regulated circRNAs (circRNA-461, circRNA-1107). miRNAs were plotted as blue nodes. **(C)** Expression pattern of the 5 DEcircRNAs between young and old rat subcutaneous adipose tissue based on mRNA sequencing. *p < 0.05, **p < 0.01 compared with young group. **(D)** GO analysis of the 5 DEcircRNAs. Using GO database (http://www.geneontology.org) analysis the GO enrichment of DEcircRNAs, based on three aspects: biological processes (BP, blue strip), cellular components (CC, red strip), and molecular functions (MF, sky blue strip). GO terms which in the red frame were related to the circRNA-1783 (circ-ATXN2). *represent the significance of the GO term enrichment among the 5 selected DEcircRNAs. *p < 0.05, **p < 0.01 compared with young group. Expression was normalized to actin and expressed as mean ± SEM (n = 3), by two-way ANOVA.

To further select the candidate circRNAs for verification, we first removed the DEcircRNAs that had no expression in any sample expression (backsplice reads = 0). Nine DEcircRNAs matched this condition. Among them, five DEcircRNAs had more than three exons. Combined with the results of the above miRNA–circRNA interactions, there were only five DEcircRNAs meeting the above three simultaneous requirements (backsplice reads of any sample were not zero, exons ≥3, and had a miRNA–circRNA interaction). The Venn diagram is shown in Figure 4A. The miRNA–circRNA interactions of the five DEcircRNAs (circRNA-967, circRNA-461, circRNA-1107, circRNA-1783, and circRNA-1176) are shown in Figure 4B. Figure 4C shows the relative expression of the five DEcircRNAs detected using RNA-seq. The GO terms of the five DEcircRNAs are shown in Figure 4D (*p* < 0.05, *p* < 0.01).

Among the above five DEcircRNAs, circ-ATXN2 (circRNA-1783), which originates from the 20 exons of ATXN2 genes, has been reported to be involved in aging-related diseases, such as amyotrophic lateral sclerosis (Liu et al., 2013; Lu et al., 2015; Tavares de Andrade et al., 2018; Zhao et al., 2018; Watanabe et al., 2020; Laffita-Mesa et al., 2021), metabolism (Carmo-Silva et al., 2017), T2D, CAD, blood pressure, and inflammation (Odegaard and Chawla 2013; Kraja et al., 2014; Carmo-Silva et al., 2017), and is associated with parental longevity and longevity (Franceschi et al., 2020). On a fat-enriched diet, ataxin-2-deficient animals showed increased weight gain (Kiehl et al., 2006). Moreover, ataxin-2 regulates the PI3K/mToR pathway, which is an important apoptotic pathway. These results suggested that ataxin-2 might act as a pro-apoptotic mediator. Thus, circ-ATXN2 was selected for further functional studies.

**FIGURE 5:**
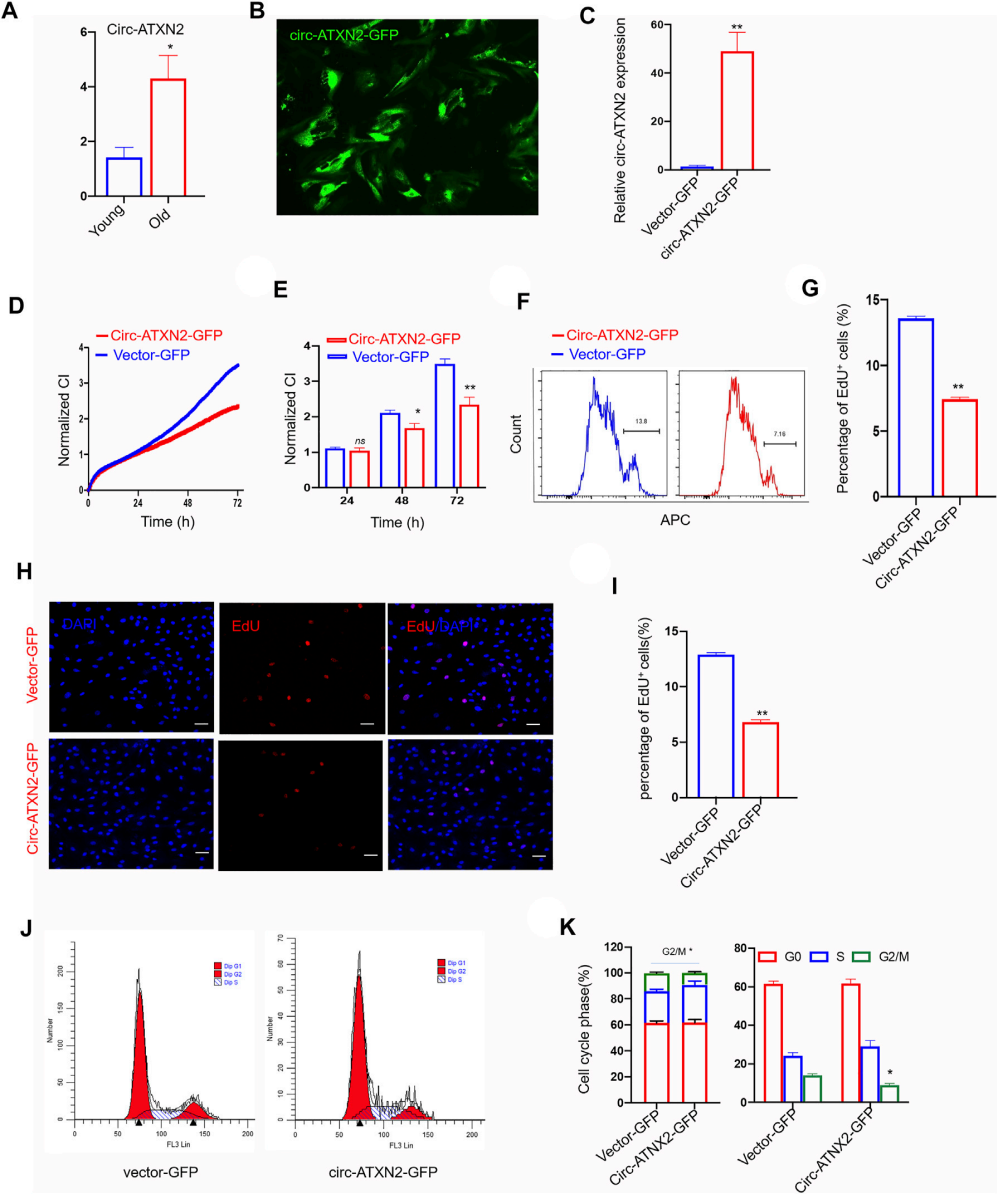
Circ-ATXN2 overexpression inhibited proliferation in rat adipose-derived stromal cells (ASCs). **(A)** The relative gene expression of circ-ATXN2 after RNase R treatment (n = 4, *p < 0.05 versus young group, statistical analysis was performed by Student’s t test). **(B)** Transfection efficiency of circ-ATNX2 based on the expression of GFP in rat ASCs observed by fluorescence microscope. **(C)** Validation of circ-ATNX2 over-expression-vector in rat ASCs by qPCR. Results were presented as mean ± SEM, by Student t test, n = 6; **p < 0.01. Cell proliferation was accessed by xCELLigence real-time cell analysis (RTCA) system assay **(D–E)** and EdU incorporation assay **(F–I)**. D. Cell proliferation was assessed using RTCA for 72 h. Statistics at 24, 48 and 72 h(E). Cell Index (CI) is an arbitrary unit reflecting the electronic cell-sensor impedance. Results were presented as mean ± SEM, by two-way ANOVA, n = 3; *p < 0.05, **p < 0.01. F. Representative images of cell proliferation were assayed using EdU incorporation by flow cytometry. **(G)** Statistics results were presented as mean ± SEM, by Student t test, n = 3; **p < 0.01. **(H)** Representative fluorescence microscope images of cell proliferation was assayed using EdU incorporation. EdU positive cell was red. DAPI was blue. Scale bars: 20 µm. **(I)** Statistics data of EdU assay were presented as mean ± SEM, by Student t test, n = 3; **p < 0.01. Cell phases of the circ-ATXN2 overexpression cells were analyzed by flow cytometry **(J)** and statistics **(K)**. Results were presented as mean ± SEM, by Student’s *t* test, n = 3; *p < 0.05. ns: not significant.

Thereafter, we demonstrated that circ-ATXN2 has a circular structure. Total RNA was treated with exoribonuclease RNase R, which is known to selectively degrade linear RNA rather than circRNAs (Jeck et al., 2013; Memczak et al., 2013). RT-PCR showed that circ-ATXN2 was upregulated during aging in the SAT of rats (*p* < 0.01, Figure 5A). Moreover, this finding was consistent with the RNA-seq result that demonstrated significantly upregulated circ-ATXN2 expression in old rats (Figure 2C). In summary, our data showed that circ-ATXN2 is a type of circRNA that exists stably and abundantly in rat SAT and that the intrinsic expression levels were upregulated in rat SAT during aging.

### Overexpression of Circ-ATXN2 Inhibited Proliferation of ASCs

We explored the effect of circ-ATXN2 on the proliferation of ASCs. We overexpressed circ-ATXN2 in ASCs using the pLC5-ciR-GFP vector. The circ-ATXN2 expression level was significantly enhanced in circ-ATXN2–GFP-expressing cells (Figures 5B,C). Vector-GFP-expressing cells were used as controls. The RTCA system assay demonstrated that the circ-ATXN2–GFP-expressing cells had a lower CI index than vector-GFP-expressing cells (*p* < 0.05, *p* < 0.01, Figure 5D). The statistical data showed that proliferation significantly decreased from 48 h after culture (Figure 5E). Using flow cytometry and immunofluorescence, we further validated that circ-ATXN2 overexpression could inhibit proliferation using EdU incorporation assays (Figures 5F,H). The EdU-positive rate decreased from 13.6 ± 0.21% to 7.43 ± 0.20%, as determined using the flow cytometry assay (*p* < 0.01, Figure 5G). The EdU-positive rate decreased from 12.9 ± 0.29% to 6.8 ± 0.33%, as determined using immunofluorescence assay (*p* < 0.01, Figure 5I). Cell cycle analysis revealed that the proportion of cells in the G0/G1 phase of circ-ATXN2–GFP-expressing cells was increased, whereas the proportion of cells in the G2 phase decreased (*p* < 0.05, Figures 5J,K). These results indicated that circ-ATXN2 inhibited proliferation.

### Overexpression of Circ-ATXN2 Promoted Cell Death of ASCs

**FIGURE 6:**
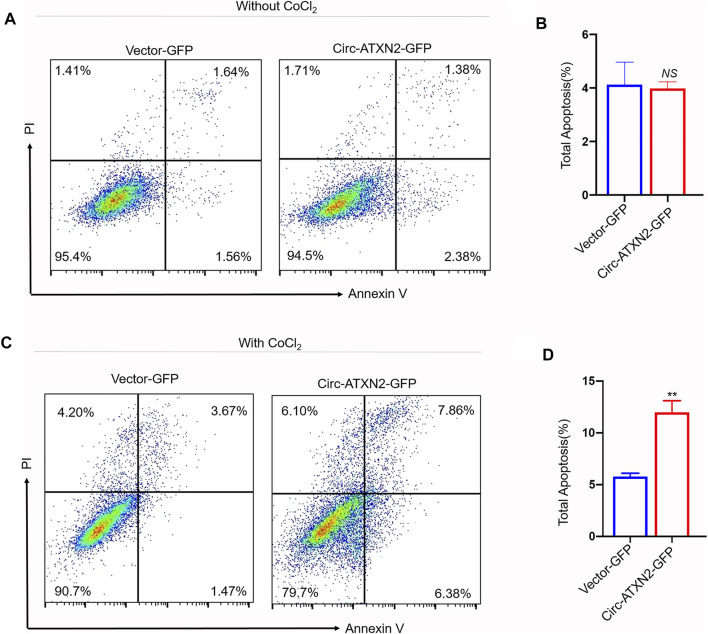
Overexpression of circ-ATXN2 enhanced apoptosis in rat adipose-derived stem cells (ASCs). Cell apoptosis was determined by Annexin V-APC/PI counterstain followed by flow cytometry. **(A)** Apoptosis, detected using flow cytometry, showed the apoptotic cell rate in vector-GFP or circ-ATXN2-GFP-expressing cells. ASCs were without treated by CoCl_2_. **(B)** Data from experiments performed at least in triplicate are presented, showing the percentage of total apoptosis. **(C)** Apoptosis assay detected by flow cytometry showed the apoptotic cell rate in vector-GFP or circ-ATXN2-GFP cells. ASCs were treated by CoCl_2_ (500 μM, 24 h). **(D)** Data from experiments performed at least in triplicate are presented, showing the percentage of total apoptosis percentage. Results were presented as means ± SEM, by Student’s *t* test, n = 3; **p < 0.01, *NS*: no significance *vs* vector-GFP-expressing cells.

To reveal the role of circ-ATXN2 in cell death, CoCl_2_ was used to induce apoptosis (Figure 6A). In circ-ATXN2–GFP-expressing cells, the total apoptotic rate was not significant different in rat ASCs (Figure 6B). However, the circ-ATXN2–GFP-expressing cells were treated with 500 µM CoCl_2_ for 24 h and assessed using flow cytometry (Figure 6C). In circ-ATXN2–GFP-expressing cells, the total apoptotic rate increased from 5.78 ± 0.46% to 11.97 ± 1.61% (*p* < 0.01) (Figure 6D), early apoptotic rate increased from 1.76 ± 0.22% to 5.50 ± 0.66% (*p* < 0.01), and late apoptosis rate increased from 4.02 ± 0.25% to 6.47 ± 1.06% (*p* < 0.01) in rat ASCs, establishing the fact that circ-ATXN2 promoted cell death in ASCs.

### Overexpression of Circ-ATXN2 Promoted Adipogenesis in ASCs

**FIGURE 7:**
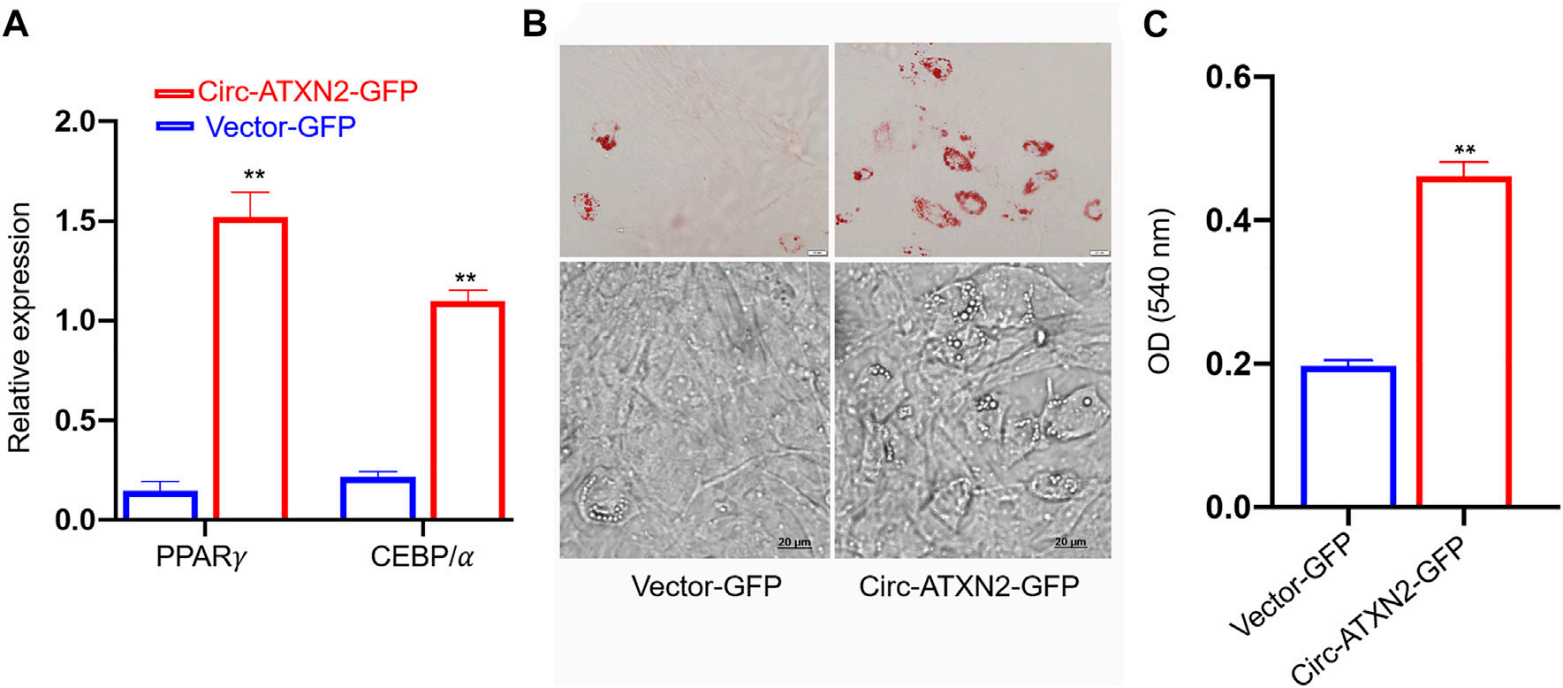
Overexpression of circ-ATXN2 promoted adipogenic differentiation in rat adipose-derived stromal cells (ASCs). **(A)** The relative mRNA level of adipocyte specific genes including *PPARγ* and *CEBP/α* were measured at 48 hours of adipogenic differentiation. Results were presented as mean ± SEM, by two-way ANOVA, n = 3; **p < 0.01, *vs* vector-GFP cells. **(B)** Representative microscope images of differentiated adipocytes in vector-GFP and circ-ATXN2-GFP cells at day 14 of adipogenic differentiation. **(C)** Quantification of Oil Red O staining results by measuring the corresponding absorbance dye at 540 nm. Results were presented as means ± SEM, n = 3; **p < 0.01, by Student *t* test, *vs* vector-GFP cells.

The relative expression of adipose differentiation-related genes *PPARγ* and *CEBP/α* was significantly increased in circ–ATXN2-GFP-expressing cells (*p* < 0.01, Figure 7A). The Oil Red O staining assay results showed more lipid droplets in circ-ATXN2–GFP-expressing cells than in the vector-GFP-expressing cells (*p* < 0.01, Figures 7B,C). These results demonstrated that circ-ATXN2 promoted adipogenesis in ASCs.

## Discussion

### Key Findings

This study is the first to reveal the presence of age-related circRNAs in rat adipose tissue. A total of 4,860 and 4,952 circRNAs was detected in 10-week-old and 27-month-old rats, respectively. Sixty-seven circRNAs were DEcircRNAs (fold change ≥2, *p* ≤ 0.05). In primary cultured ASCs, overexpression of circ-ATXN2 significantly decreased the proliferation and increased the cell death. Furthermore, overexpression of circ-ATXN2 increased the expression levels of adipose differentiation-related genes and promoted adipogenesis.

### The Relationship With Previous Studies

Similar to the studies of adipose tissue in aging (Bonzon-Kulichenko et al., 2018; Liu et al., 2018), H and E staining showed that the diameter of adipocytes was increased in adipocytes from old rats, although the number decreased. TUNEL staining showed that the percentage of dead cells was significantly increased. Previous studies have reported increased circRNA expression in the brain during aging (Gruner et al., 2016; Xu et al., 2018); however, this trend was not observed in our study in the rat SAT. Our results are consistent with previous studies on the heart and muscle tissues (Kotb Abdelmohsen et al., 2015; Gruner et al., 2016). The reasons why the expression of circRNAs in adipose tissue does not increase significantly with age are unclear and further studies are required in elucidating them.

We revealed 67 DEcircRNAs between the two groups. Thereafter, we studied the potential function of these DEcircRNAs using GO and KEGG enrichment analyses based on the expression of host genes. GO analysis showed that the top 20 significantly enriched GO terms were related to the physiological phenomena of senescent adipocytes (Karagiannides et al., 2001; Zamboni et al., 2014; Lettieri Barbato and Aquilano 2016; Stout et al., 2017), including proliferation, apoptosis, and differentiation. KEGG analysis showed that 35 DEcircRNAs had KEGG descriptions. These pathways were also related to proliferation, apoptosis, and differentiation. The main pathways included the Wnt, TGF-β, PI3K-Akt, Jak-STAT, and MAPK signaling pathways. Therefore, we believe that DEcircRNAs are involved in the processes of proliferation, cell death, and differentiation during adipose tissue aging.

We further identified five candidate circRNAs (circRNA-967, circRNA-461, circRNA-1107, circRNA-1783, and circRNA-1176) based on three filter criteria. Their characterization is shown in Figure 4 *.* Among them, we observed that the host gene of circRNA-1783 was ATXN2. ATXN2 is a large pleiotropic gene involved in various age-related diseases, including type 2 diabetes, high blood pressure, amyotrophic lateral sclerosis, and inflammation (Odegaard and Chawla 2013; Kraja et al., 2014; Laffita-Mesa et al., 2021). This discovery provides important insights. It raises the question: whether circRNA-1783 plays key roles in ASCs during aging? Stem cells can promote tissue repair and regeneration (Wang et al., 2018). It is thought that a decline in stem cell function leads to aging. We found that the expression level of circ-ATXN2 was increased during adipose tissue aging. Thereafter, we analyzed the role of circ-ATNX2 in the proliferation, cell death, and adipogenesis of ASCs.

Researchers have made significant efforts to find mechanisms that can keep stem cells young, such as the mitochondrial unfolded protein response (Shen et al., 2020). However, it is also important to identify a mechanism that can induce stem cell senescence. We demonstrated that overexpression of circ-ATXN2 reduced the proliferation of ASCs and promoted their cell death and adipogenesis, which was similar to the recent reports of hsa_circRNA_0001776 and circRNA_100,876 (Jin et al., 2019; Jia et al., 2020). By contrast, we noticed that some circRNAs had opposite effects. For example, circRNA-0008717 promotes cell proliferation (Wang et al., 2020) and circRNA.7079 has anti-apoptotic effects (Yao et al., 2020), whereas circular RNA H19 inhibits adipogenesis in human ASCs. We speculate that different types of circRNAs may play different roles in different organizations and animals. When comparing our study to their research, we found that their reports mostly focused only on one or two functions of the target circRNA. This study analyzed the functions of circ-ATNX2, including proliferation, cell death, and adipogenesis.

### Potential Limitations

This study revealed the aging-related function of circ-ATNX2; however, this study has several limitations. First, we only studied circ-ATNX2, one of the five candidate circRNAs, and no further study of the downstream mechanism of circ-ATNX2 was conducted. However, based on the GO and KEGG analyses, we obtained clues for miRNA–circRNA interactions. CircRNA, as an miRNA sponge, indirectly interferes with mRNA translation, gene shearing, and transcription, and interacts with RNA binding proteins, thus affecting the regulation of gene function (Guo et al., 2020; Qi et al., 2021). Candidate miRNAs are shown in Figure 4B. These candidate miRNAs and their related genes are available for future studies. Second, we are aware of the limitations of the conclusions drawn from an aging trend with only two-time points. Future studies are needed to include more time points to detect dynamic changes in DEcircRNA expression during aging. Third, we did not explore the function of circ-ATXN2 in ASCs *in vivo*. We believe that there is an urgent need for appropriate *in vivo* study in the future that may help to gain a deeper understanding of circ-ATXN2 function in ASCs. If the location of circ-ATNX2 is clarified, it will be more conducive in understanding its mechanism. Based on our previous experience with circRNA.7079 (Yao et al., 2020), we hypothesized that circ-ATNX2 may be located in the cytoplasm or nucleus based on its functional speculation. Forth, for the cell death part of experiments *in vivo*, we applied TUNEL and immunofluorescence to detect cleaved caspase 3. Based on our results, we can only assume that cell apoptosis might have occurred in adipose tissue in aged rats. To confirm it, more methods such as TEM, IF and assessment of more caspase factors should be employed.

### Expectations

Adipose tissue may serve as an ideal target for studying fundamental aging mechanisms (Palmer and Kirkland 2016). Further investigation of the role of adipose tissue aging in ASCs functions will shed light on regenerative medicine. Therefore, adipose tissue aging is valuable for studying age-related mechanisms and is a potent therapeutic target for new therapies to combat the effects of aging and age-related diseases. Our study highlights the important role of circ-ATXN2 in regulating proliferation, cell death, and adipogenesis in ASCs. CircRNAs such as circ-ATXN2 may be a new potential target for improving the ability of ASCs for stem cell-based therapies and the ability to repair endogenous processes. Despite its preliminary character, the experimental results will hopefully serve as useful information for the studies on age-related circRNAs.

## Conclusion

Our study revealed the expression profiles of circRNAs in the adipose tissue of aged rats. We found a novel age-related circ-ATXN2 that could inhibit proliferation and promote cell death and adipogenesis in rat ASCs.

## Data Availability

The original contributions presented in the study are publicly available in NCBI using accession number PRJNA762431.

## Abbreviations

ASCs, Adipose tissue-derived stromal cells; SAT, rat subcutaneous adipose tissues; GO, Gene Ontology; KEGG, Kyoto Encyclopedia of Genes and Genomes; RTCA, real-time cell analysis; CI, Cell Index.
