# Supplementary figures and images for: A Novel Age-Related Circular RNA Circ-ATXN2 Inhibits Proliferation, Promotes Cell Death and Adipogenesis in Rat Adipose Tissue-Derived Stromal Cells
Source: Front Genet. 2021 Nov 9;12:761926. doi: 10.3389/fgene.2021.761926 (PMC8630790; doi:10.3389/fgene.2021.761926)

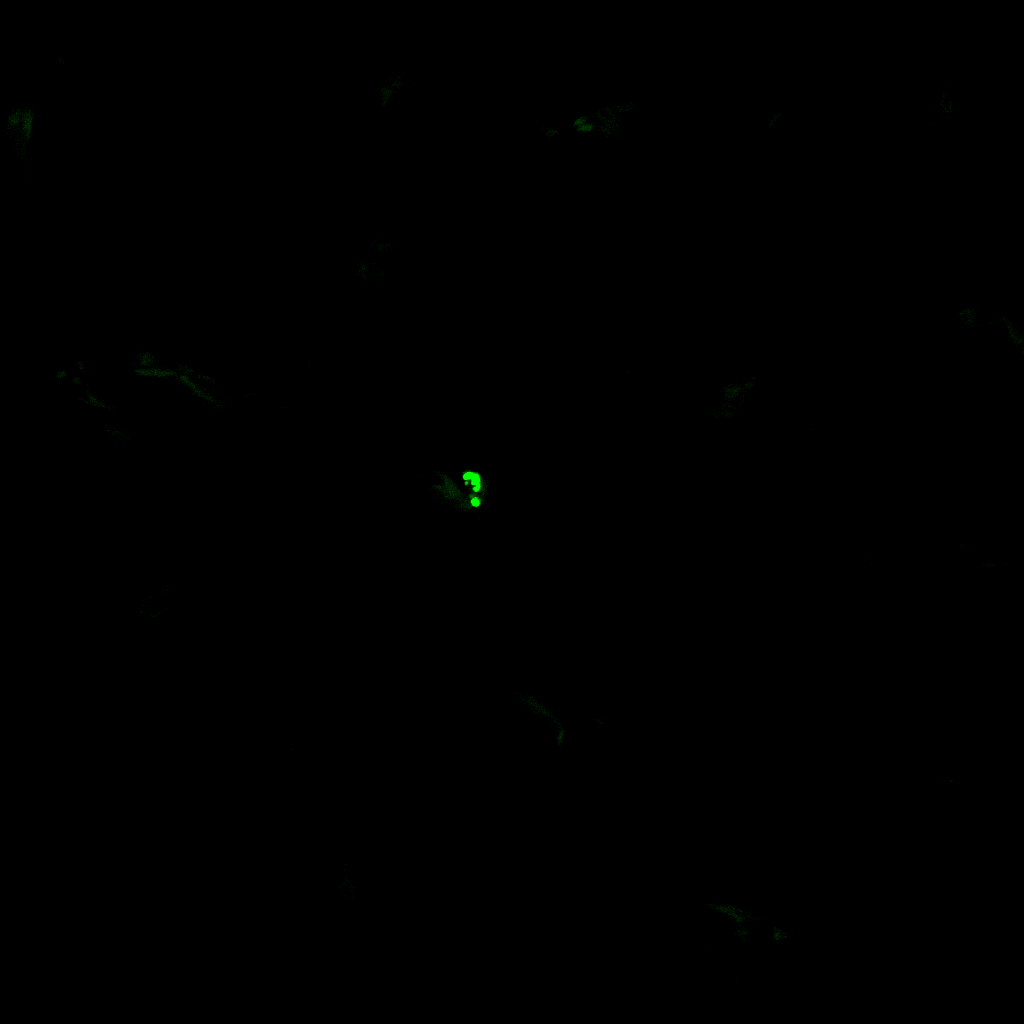

Supplement: Supplementary file 1 [file DataSheet3.ZIP › figure 1 E/young-3-2c2.tif]

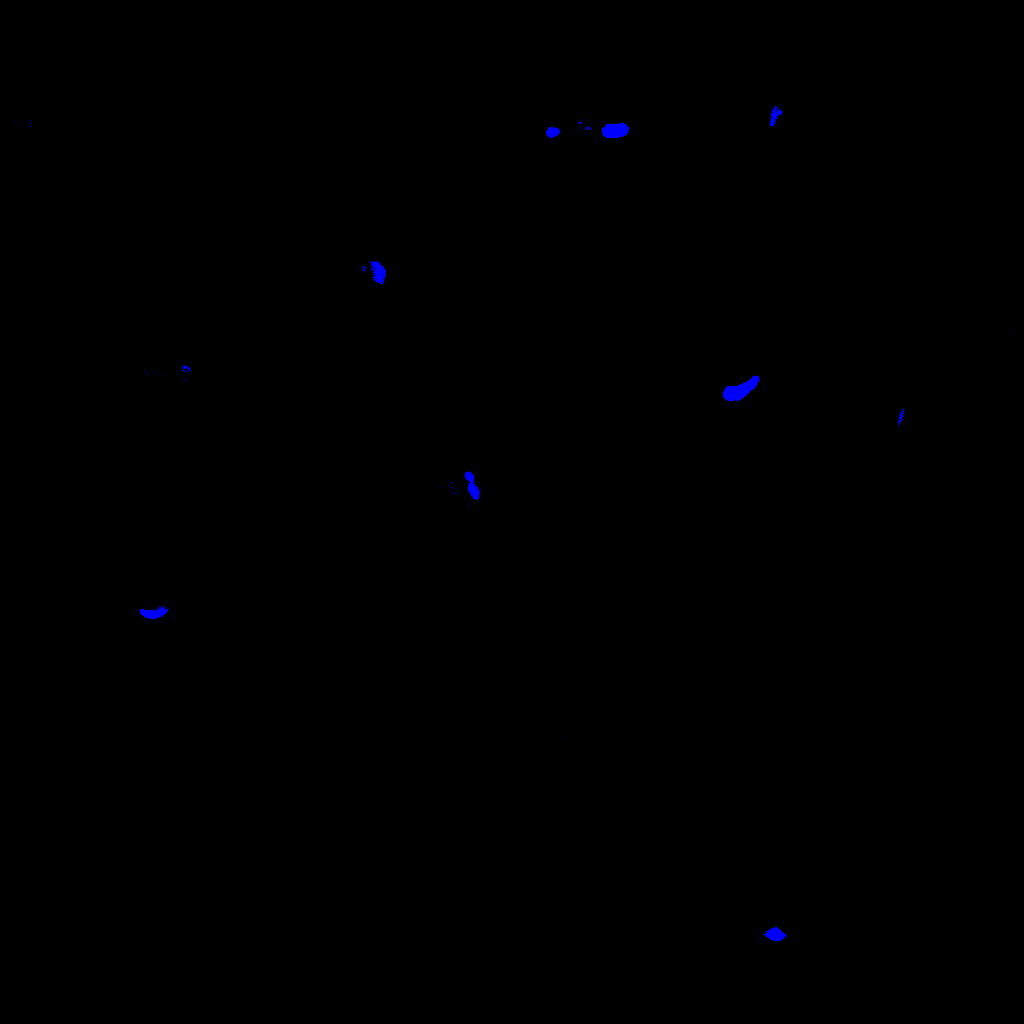

Supplement: Supplementary file 1 [file DataSheet3.ZIP › figure 1 E/young-3-2c1.tif]

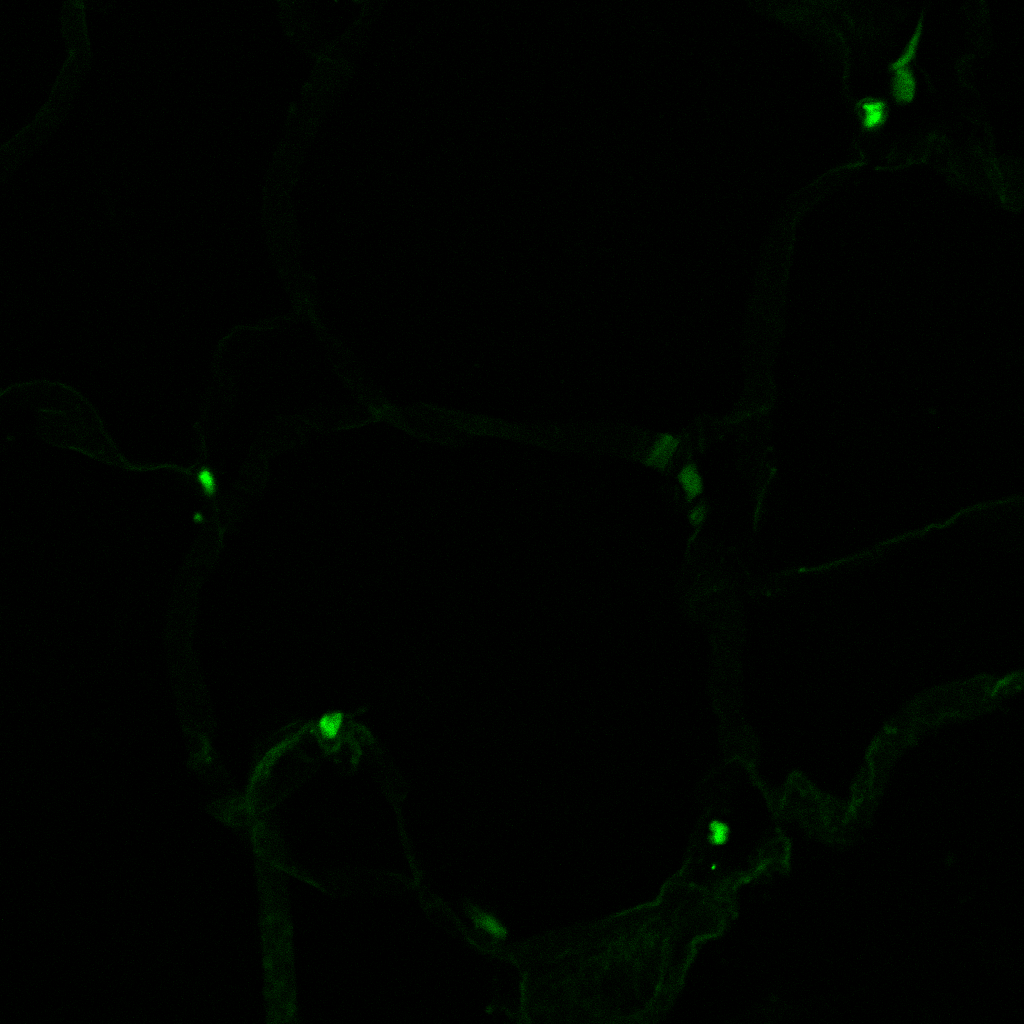

Supplement: Supplementary file 1 [file DataSheet3.ZIP › figure 1 E/old-1-2c2.tif]

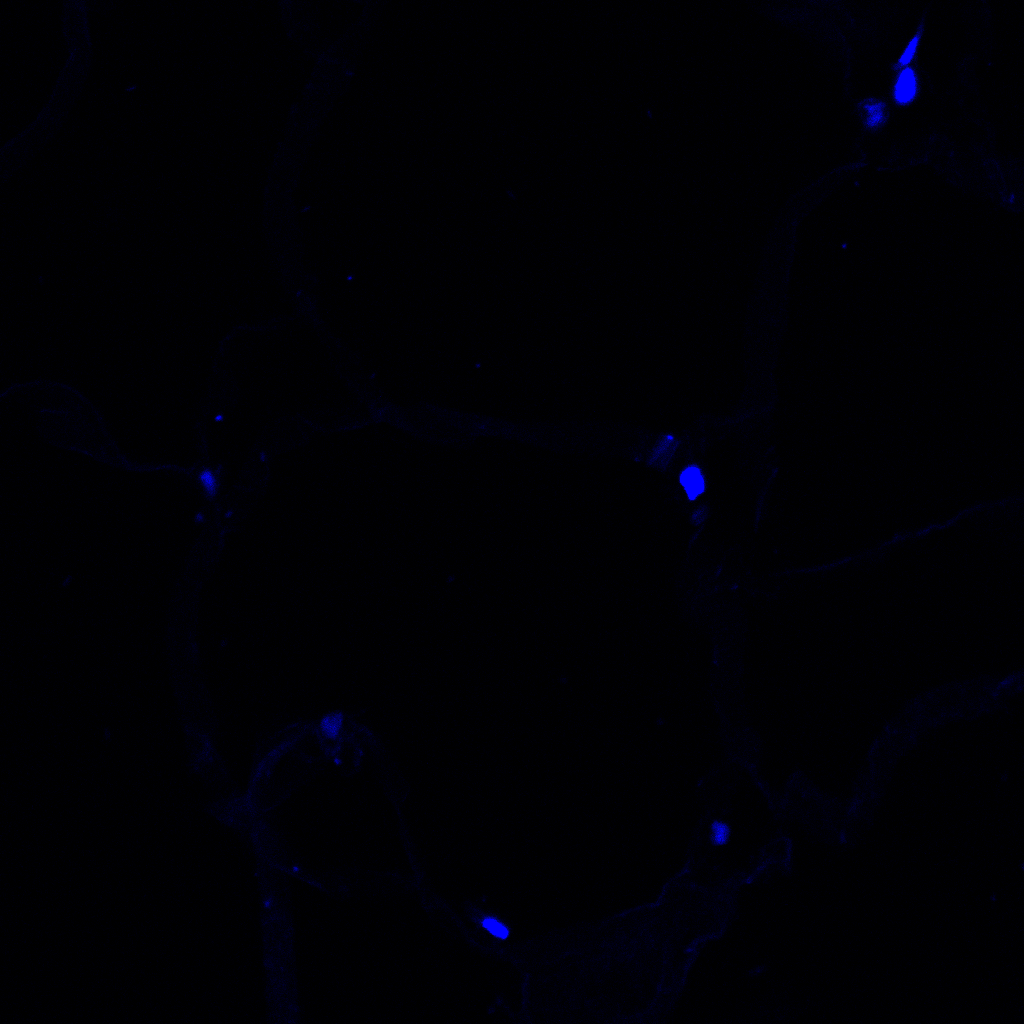

Supplement: Supplementary file 1 [file DataSheet3.ZIP › figure 1 E/old-1-2c1.tif]

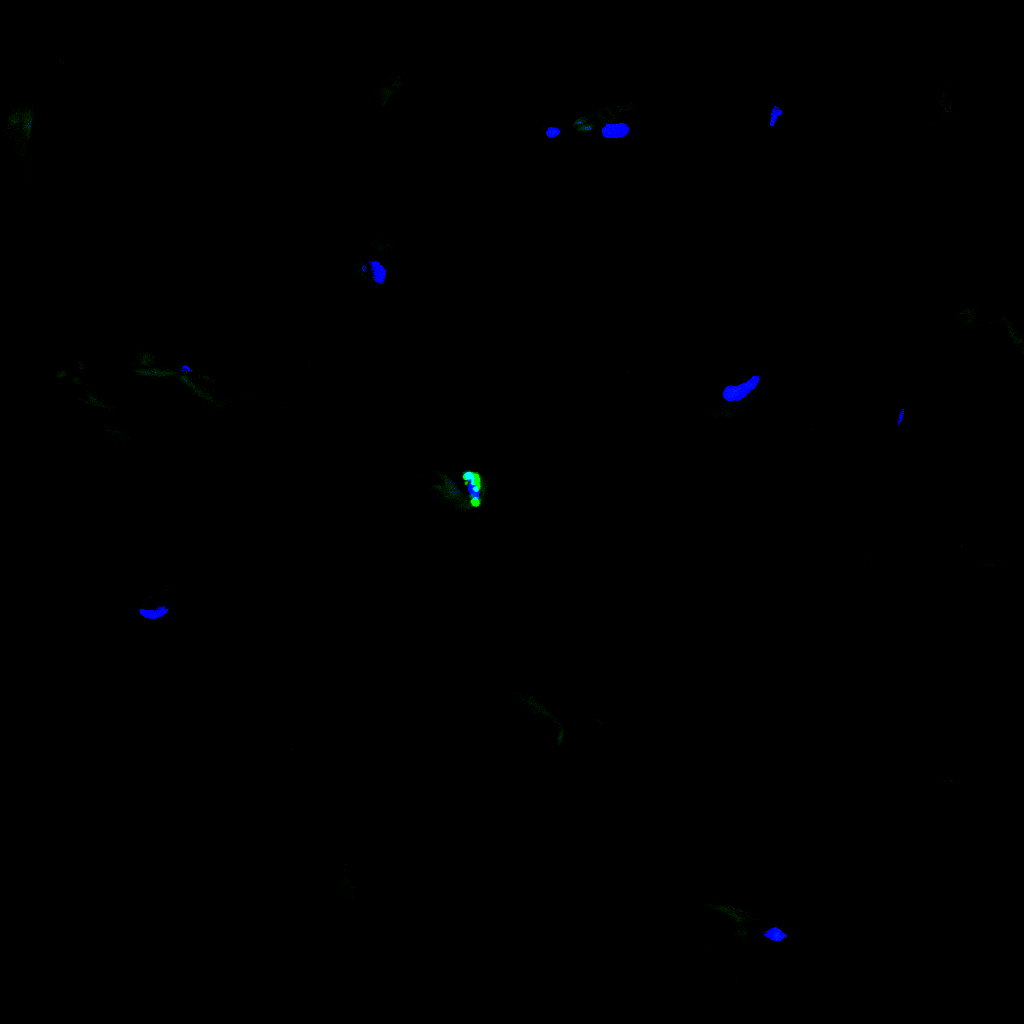

Supplement: Supplementary file 1 [file DataSheet3.ZIP › figure 1 E/young-3-2.tif]

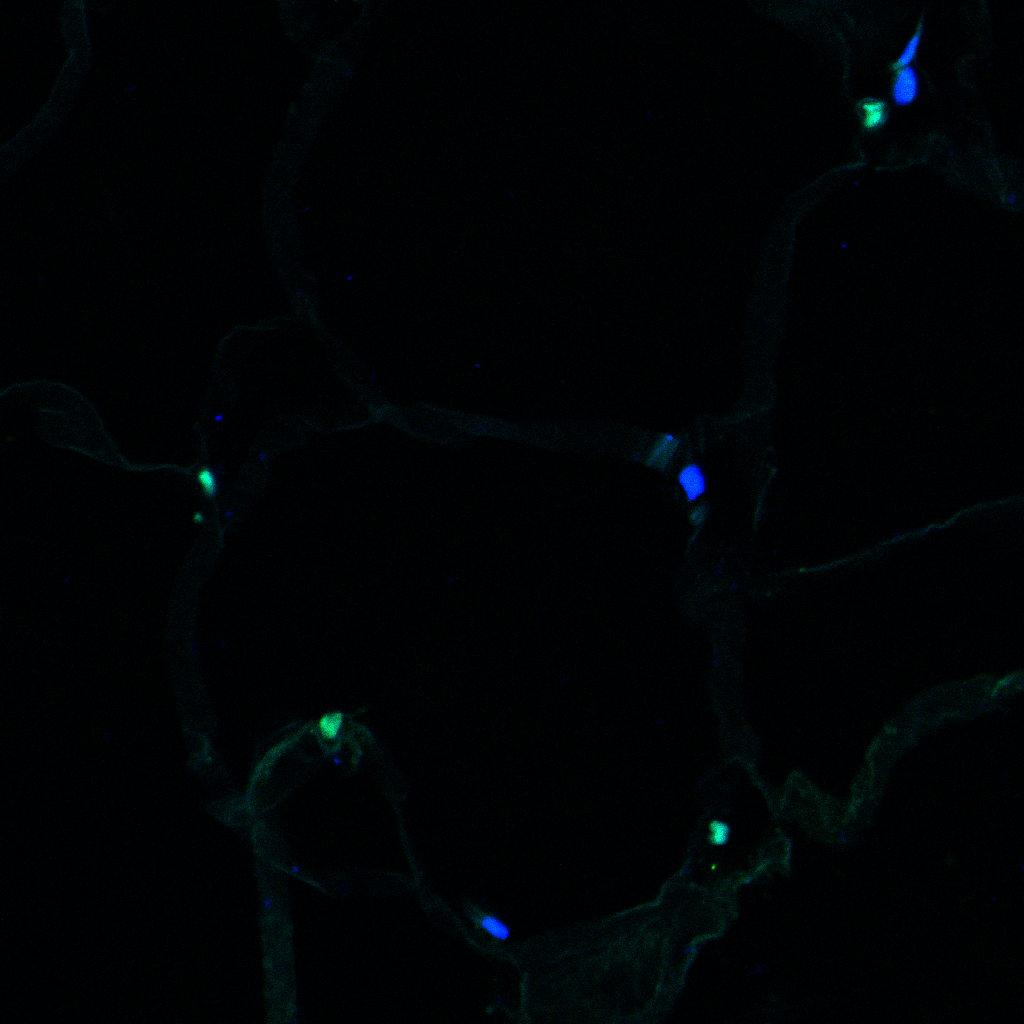

Supplement: Supplementary file 1 [file DataSheet3.ZIP › figure 1 E/old-1-2.tif]

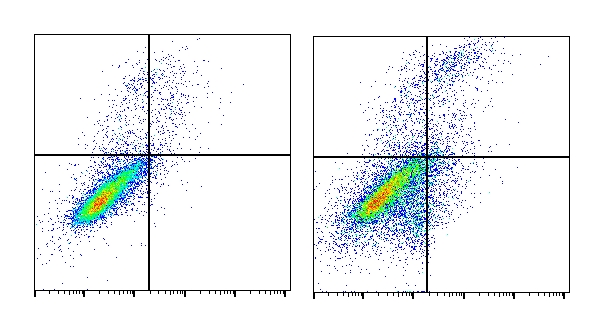

Supplement: Supplementary file 2 [file DataSheet8.ZIP › figure 6-flow cytometry apoptosis raw data/σêåμ₧É20210710-Layout2.jpg]

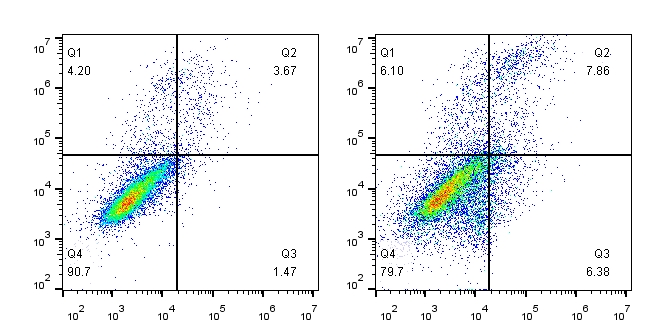

Supplement: Supplementary file 2 [file DataSheet8.ZIP › figure 6-flow cytometry apoptosis raw data/σêåμ₧É20210710-Layout.jpg]

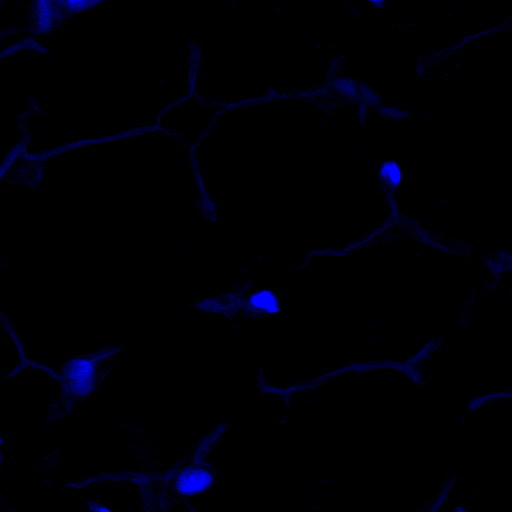

Supplement: Supplementary file 4 [file DataSheet4.ZIP › figure 1G/CON-2c1.tif]

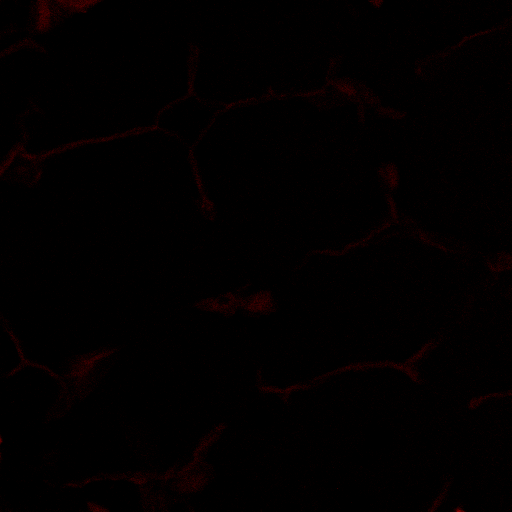

Supplement: Supplementary file 4 [file DataSheet4.ZIP › figure 1G/CON-2c2.tif]

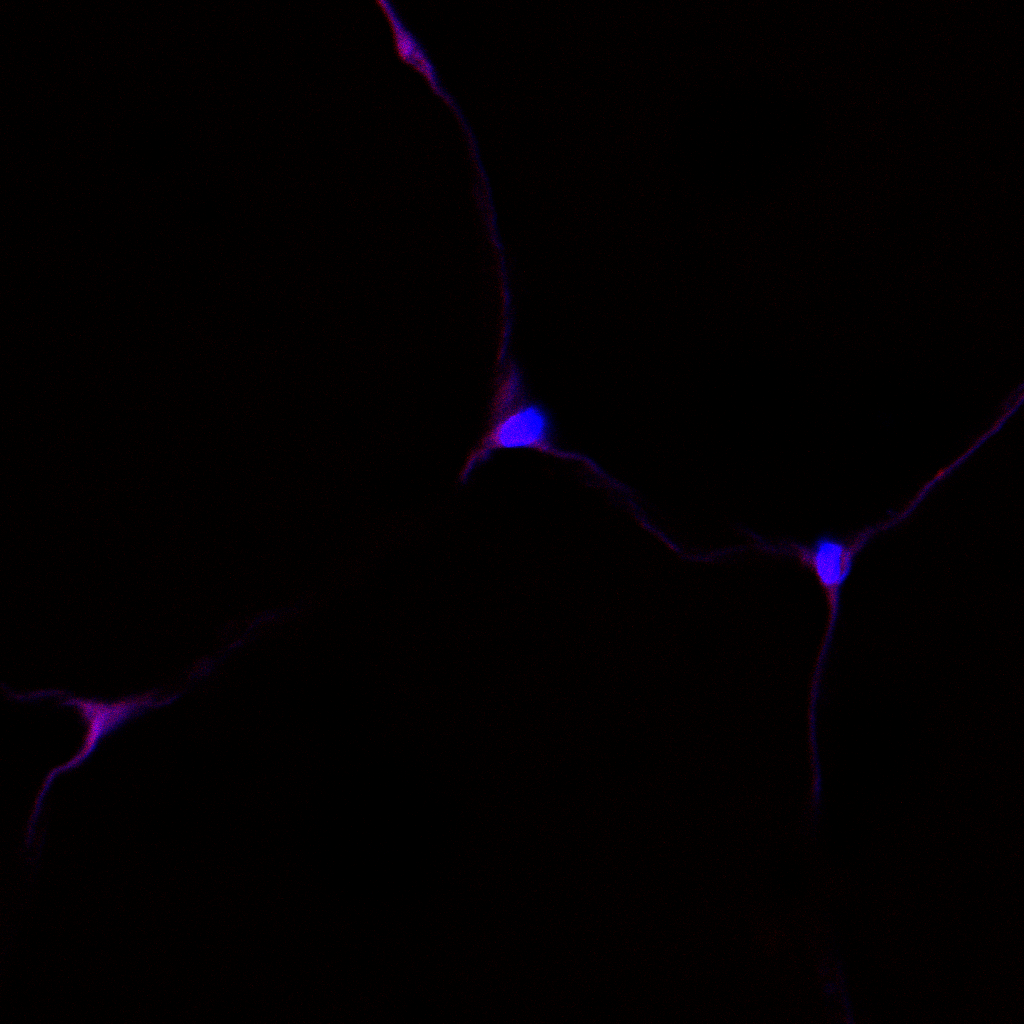

Supplement: Supplementary file 4 [file DataSheet4.ZIP › figure 1G/4.tif]

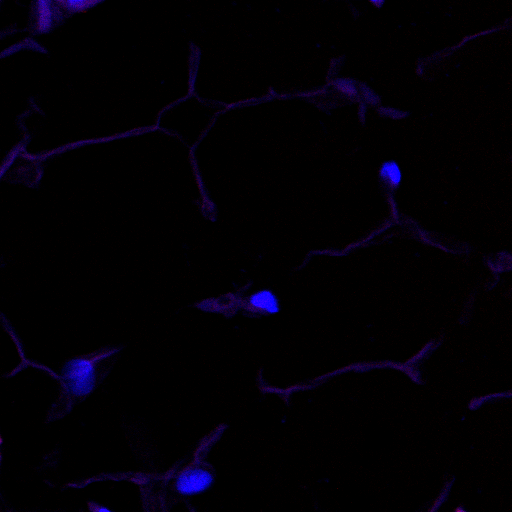

Supplement: Supplementary file 4 [file DataSheet4.ZIP › figure 1G/CON-2.tif]

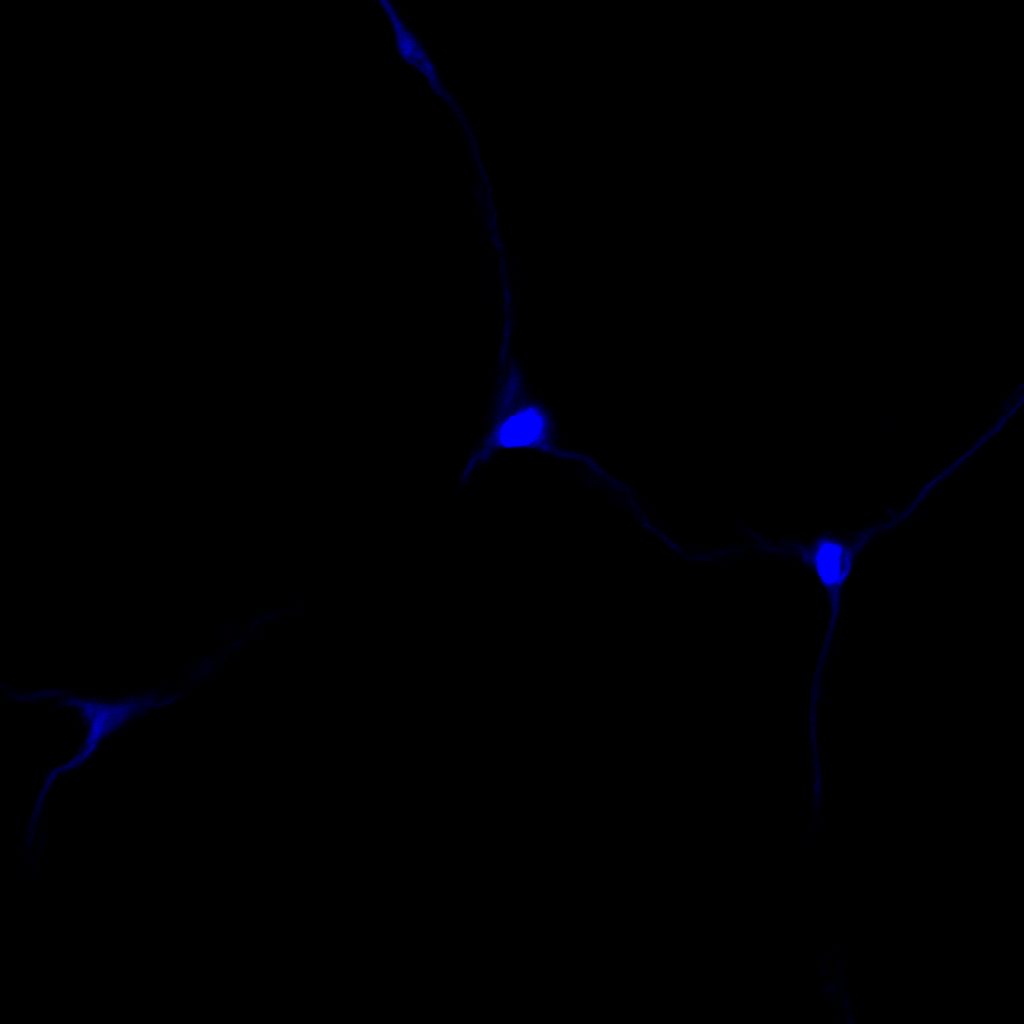

Supplement: Supplementary file 4 [file DataSheet4.ZIP › figure 1G/4c1.tif]

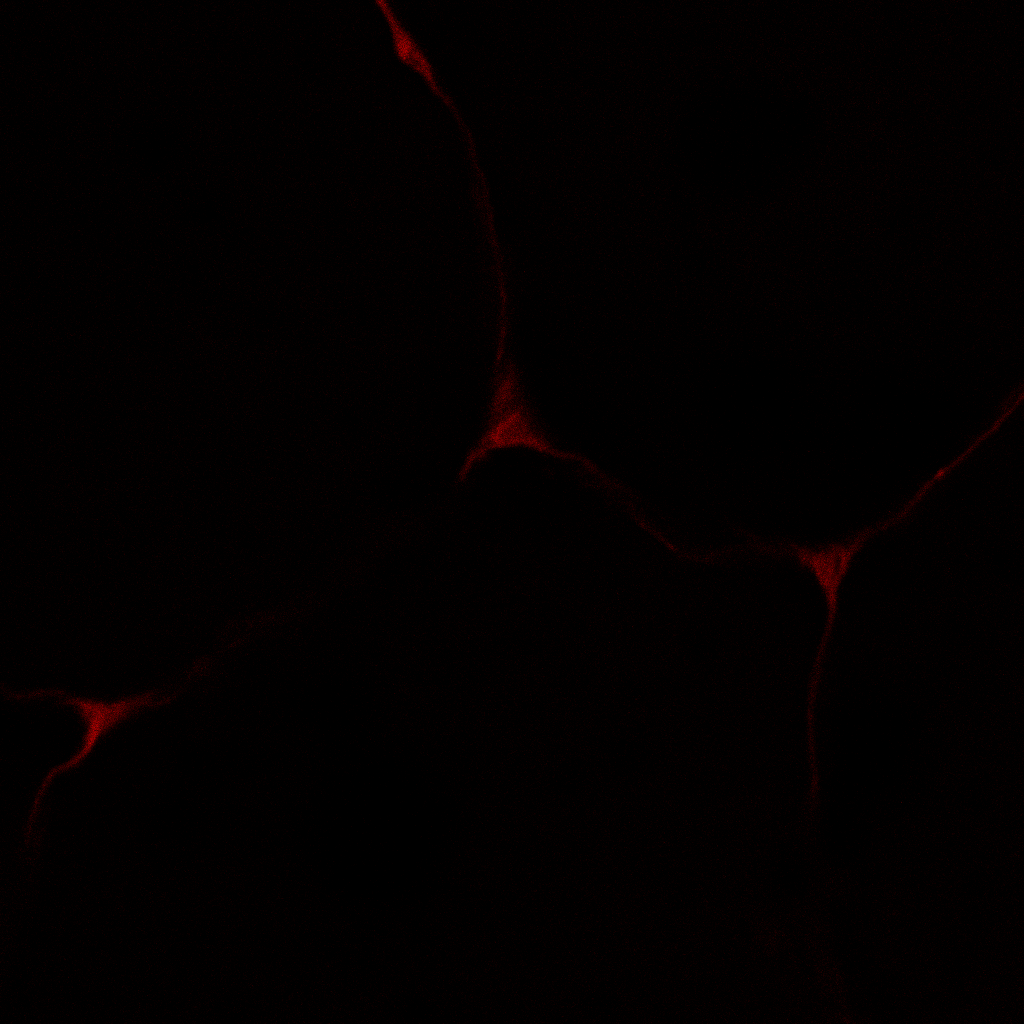

Supplement: Supplementary file 4 [file DataSheet4.ZIP › figure 1G/4c2.tif]

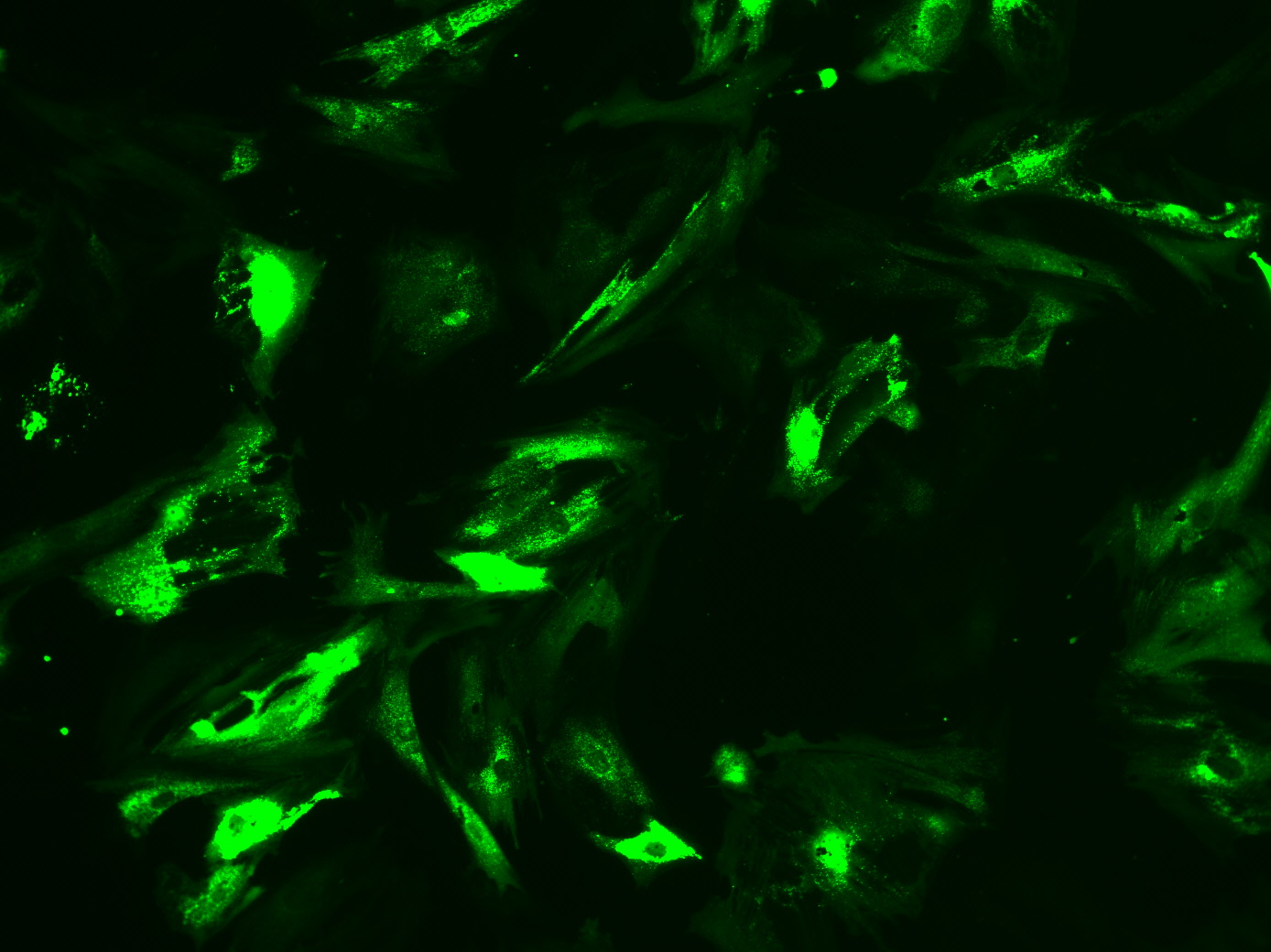

Supplement: Supplementary file 5 [file Image1.JPEG]

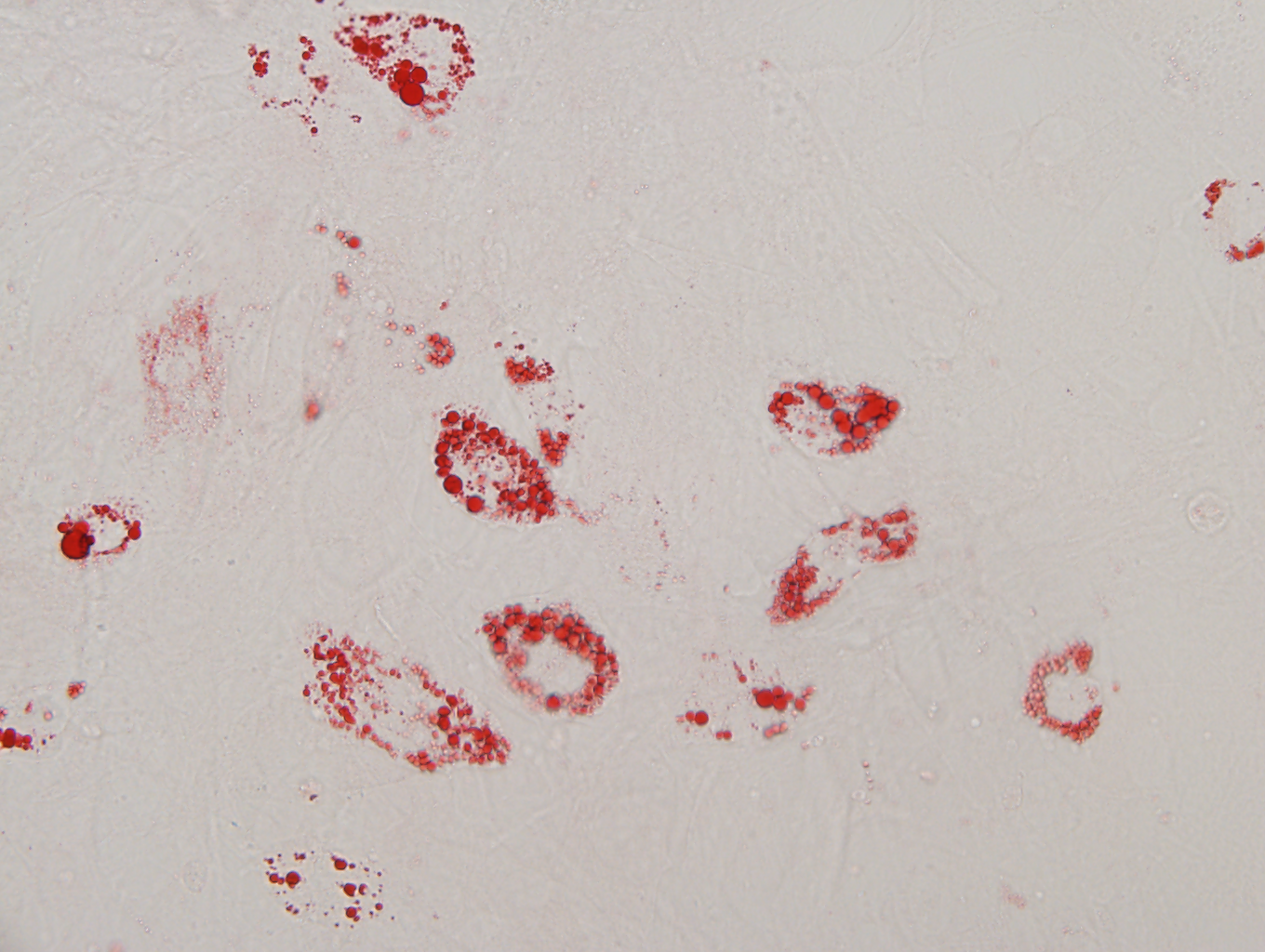

Supplement: Supplementary file 7 [file DataSheet10.ZIP › figure 7 B/circ-ATXN2-40X-2.tif]

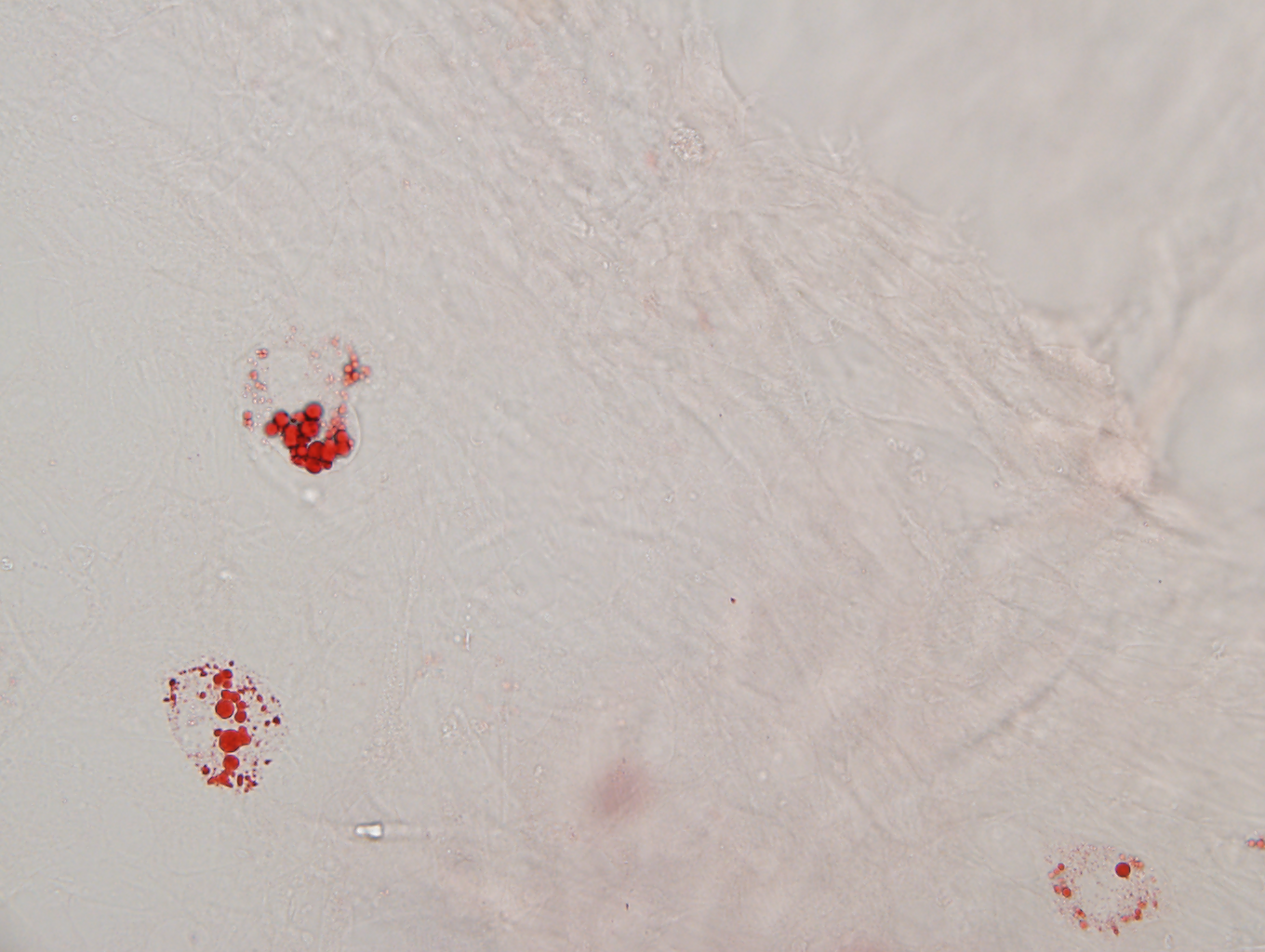

Supplement: Supplementary file 7 [file DataSheet10.ZIP › figure 7 B/Vector-40X-2.tif]

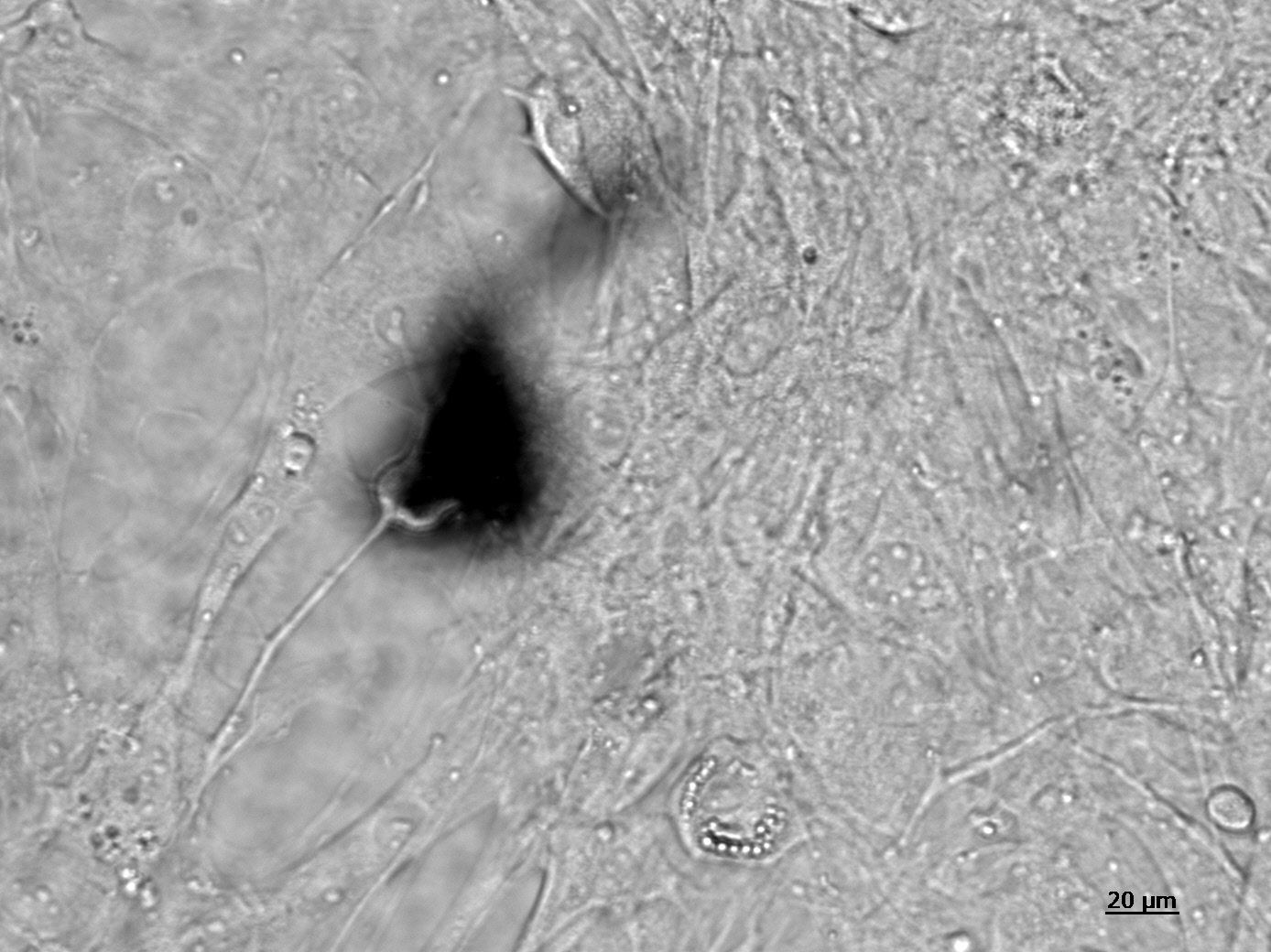

Supplement: Supplementary file 7 [file DataSheet10.ZIP › figure 7 B/figure 7 B vector-GFP.JPG]

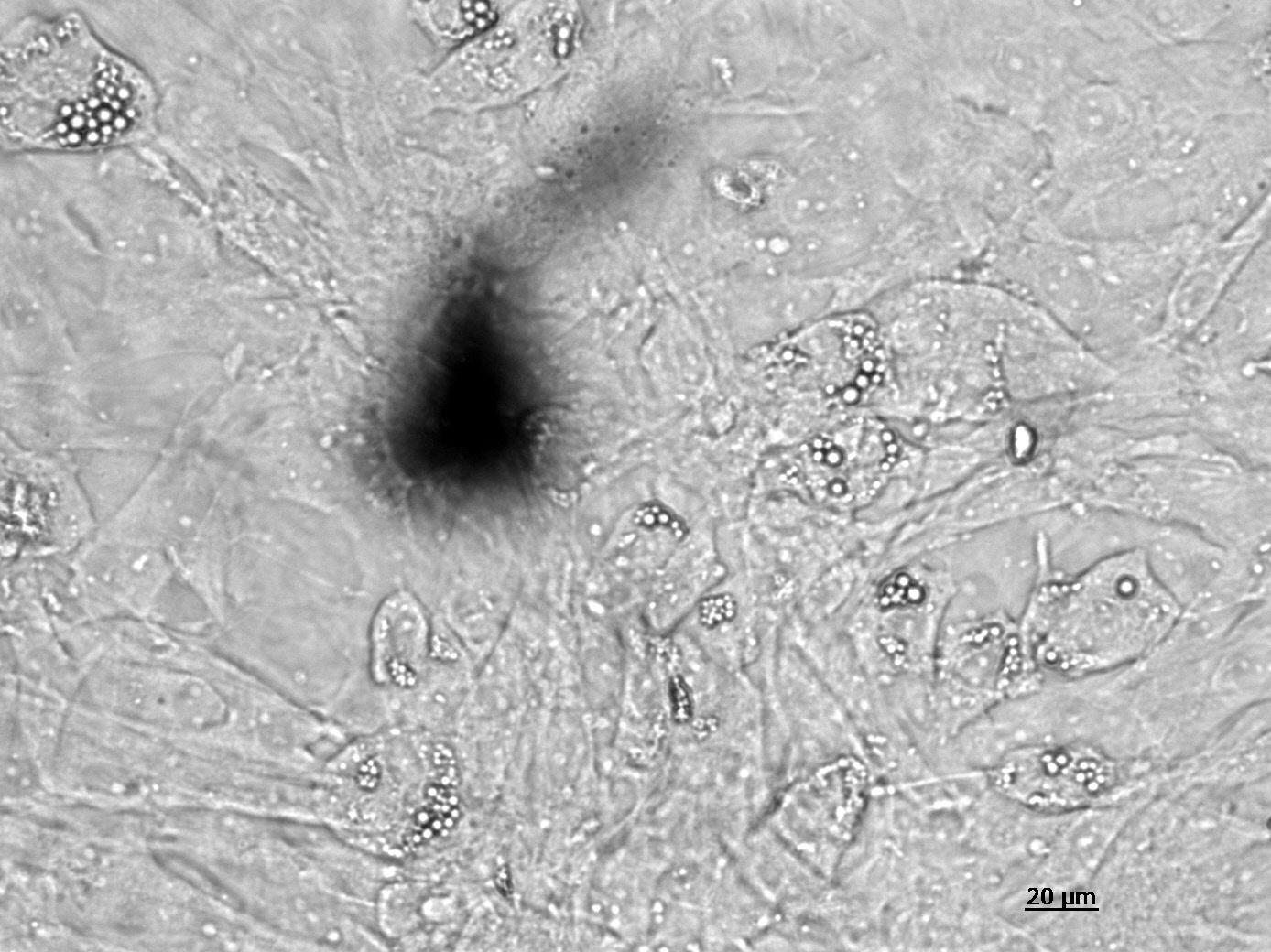

Supplement: Supplementary file 7 [file DataSheet10.ZIP › figure 7 B/figure 7 B circ-ATXN2-GFP.JPG]

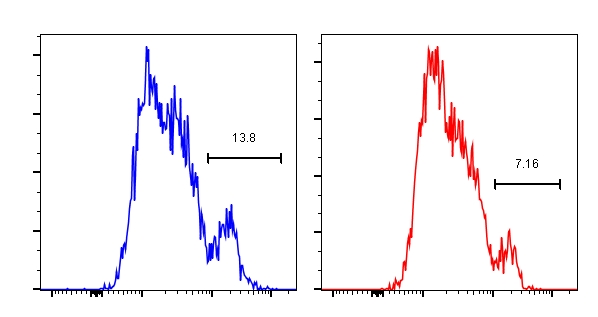

Supplement: Supplementary file 8 [file DataSheet6.ZIP › figure 5-flow cytometry edu raw data/20210622-Layout.jpg]

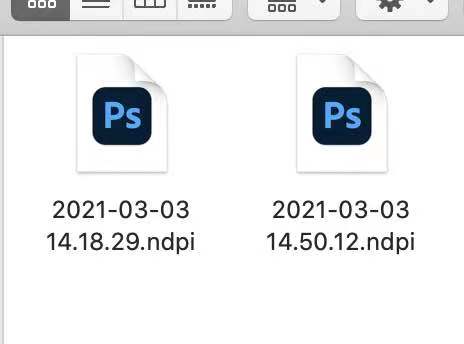

Supplement: Supplementary file 9 [file DataSheet2.ZIP › figure 1 A/WechatIMG48.jpeg]

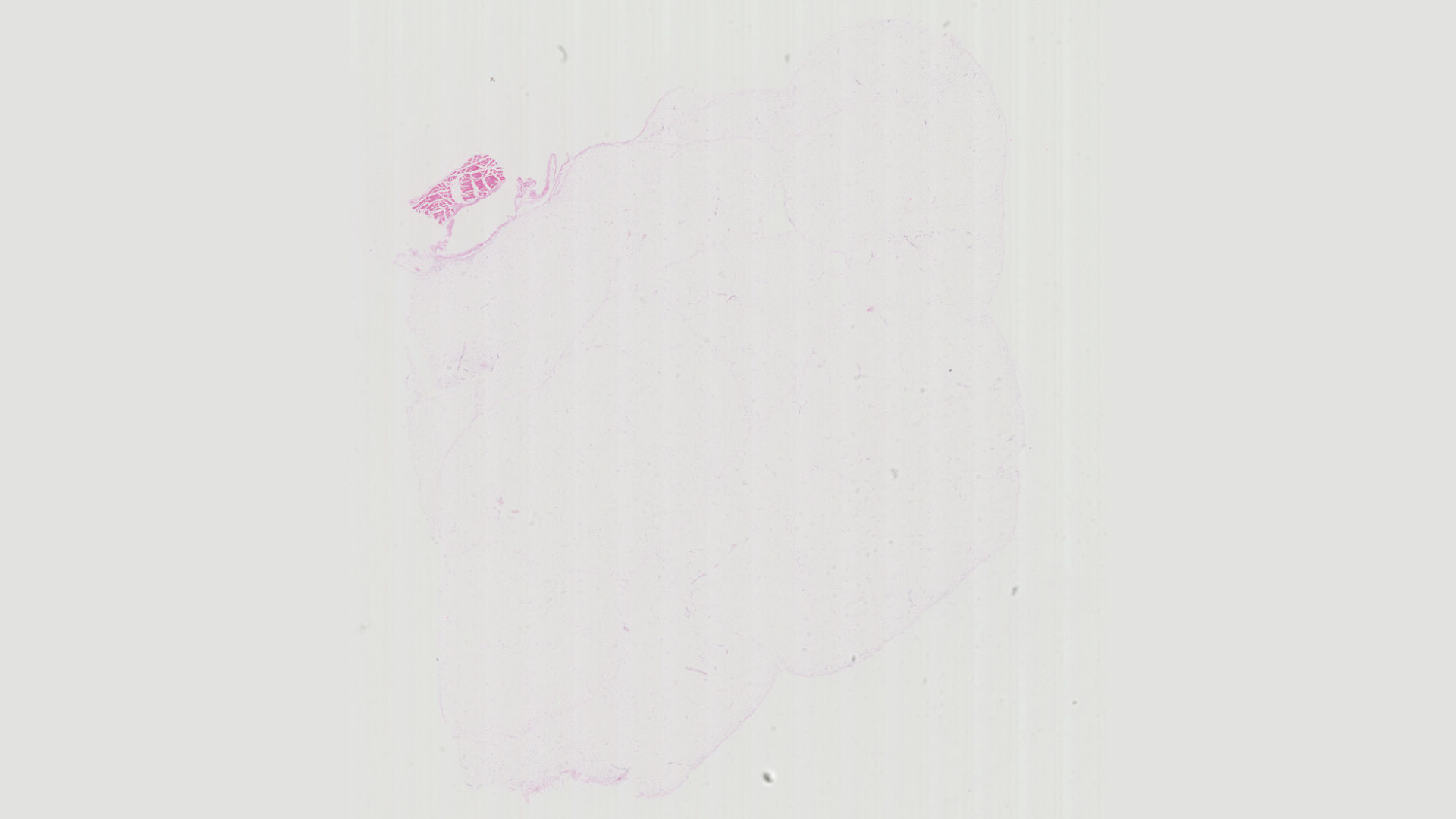

Supplement: Supplementary file 9 [file DataSheet2.ZIP › figure 1 A/old-adipose tissue.jpg]

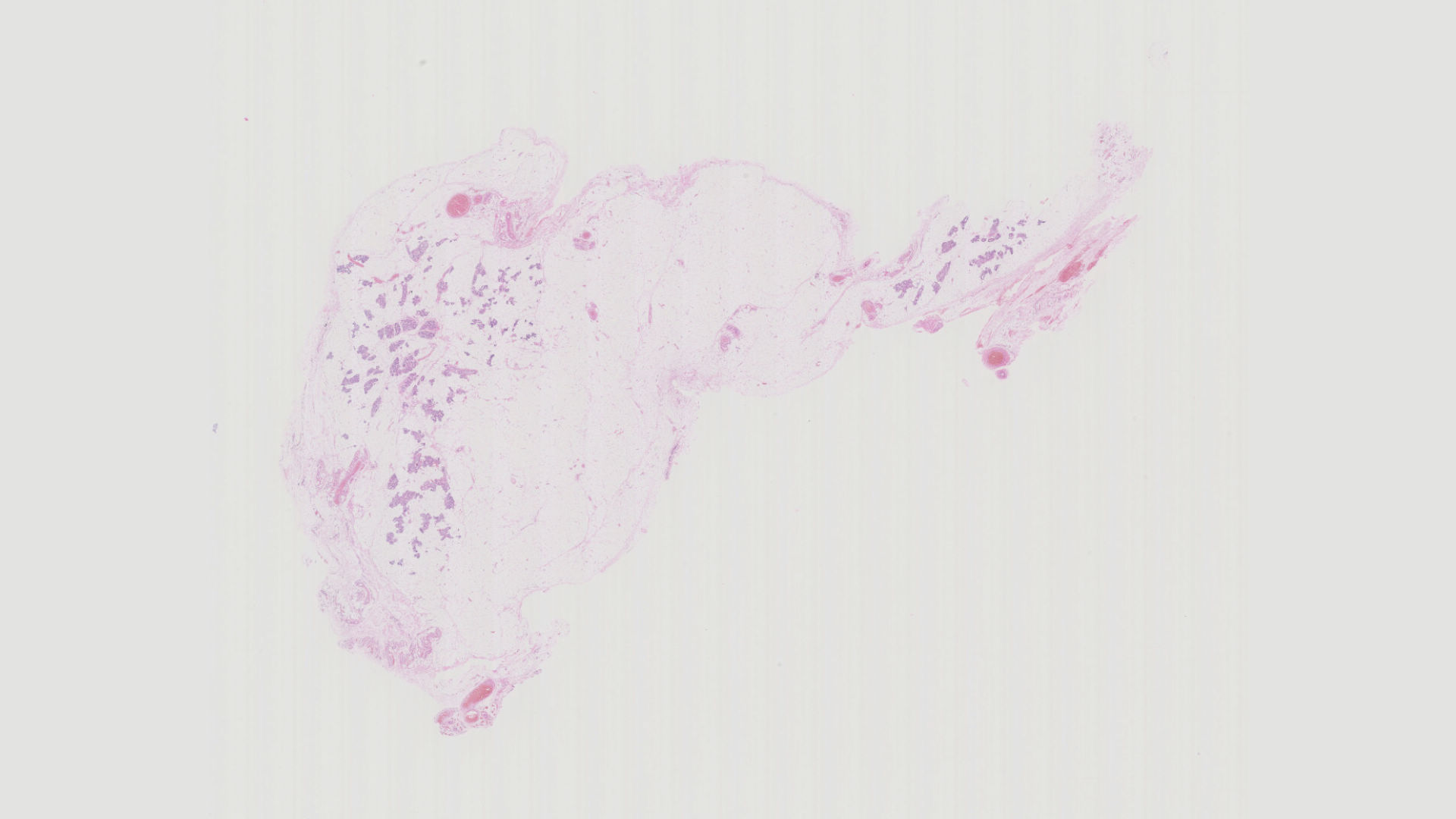

Supplement: Supplementary file 9 [file DataSheet2.ZIP › figure 1 A/young-adipose tissue.jpg]

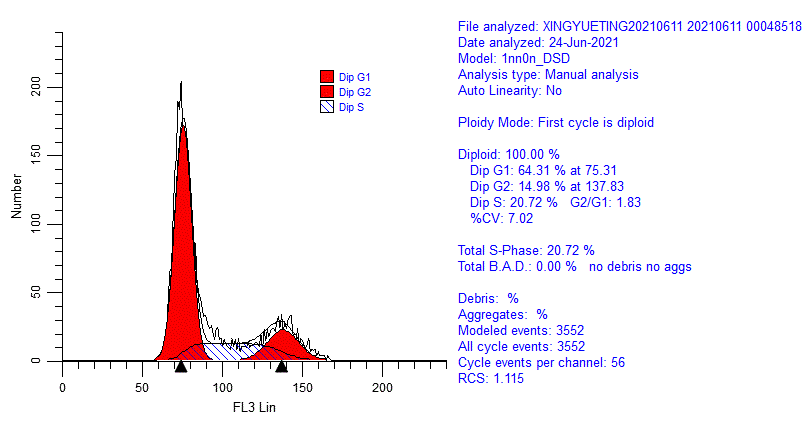

Supplement: Supplementary file 10 [file DataSheet5.ZIP › figure 5 -flow cytometry raw data/518.GIF]

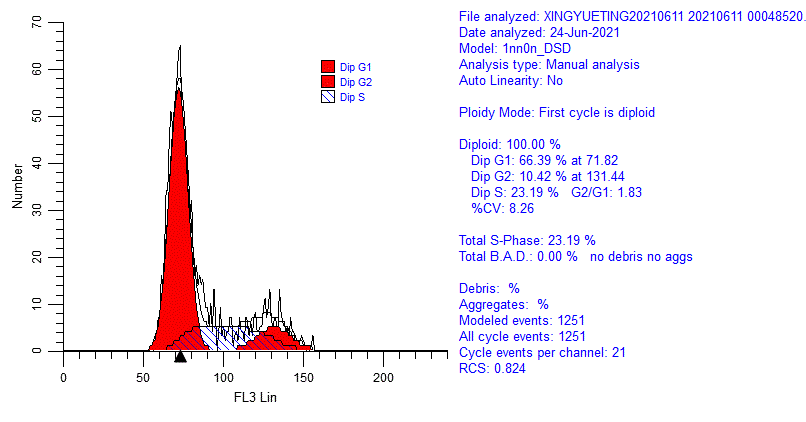

Supplement: Supplementary file 10 [file DataSheet5.ZIP › figure 5 -flow cytometry raw data/520.GIF]

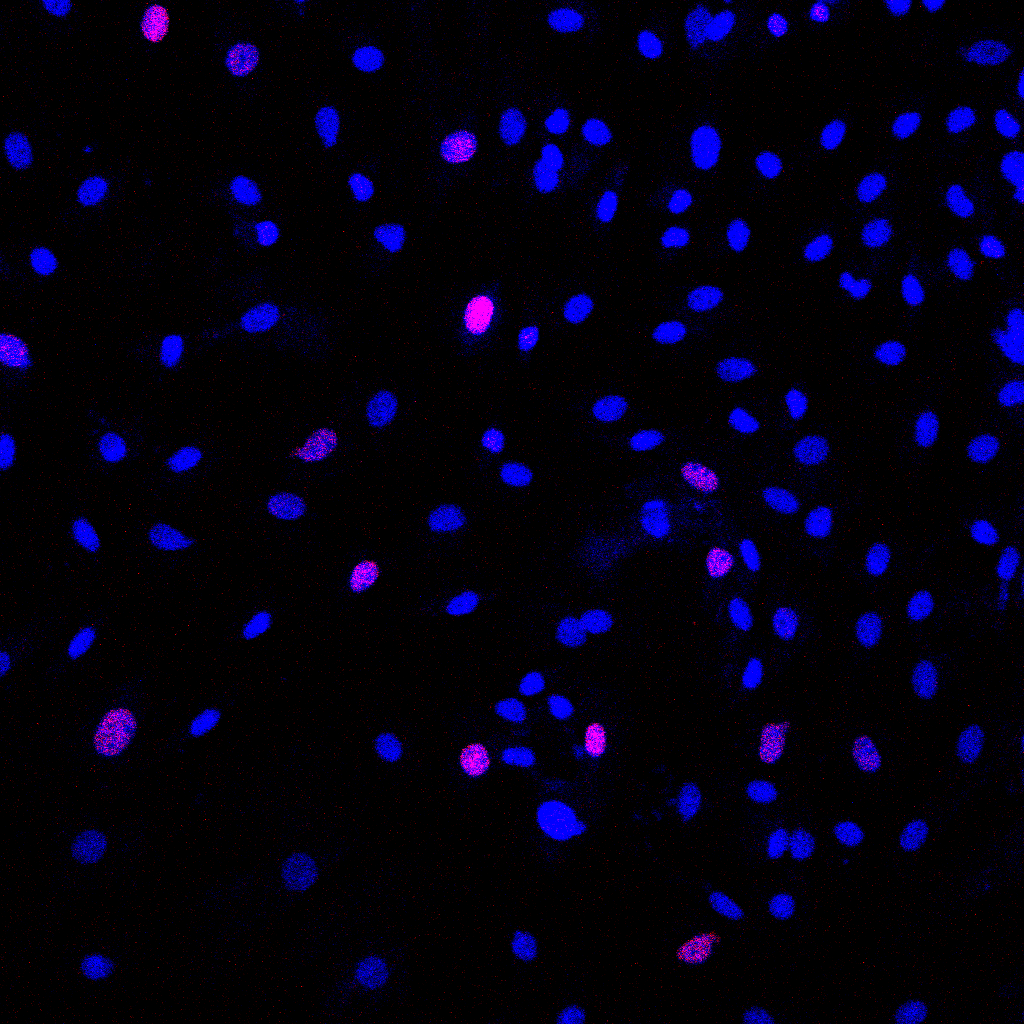

Supplement: Supplementary file 11 [file DataSheet7.ZIP › figure 5 H/VECTOR-Composite.tif]

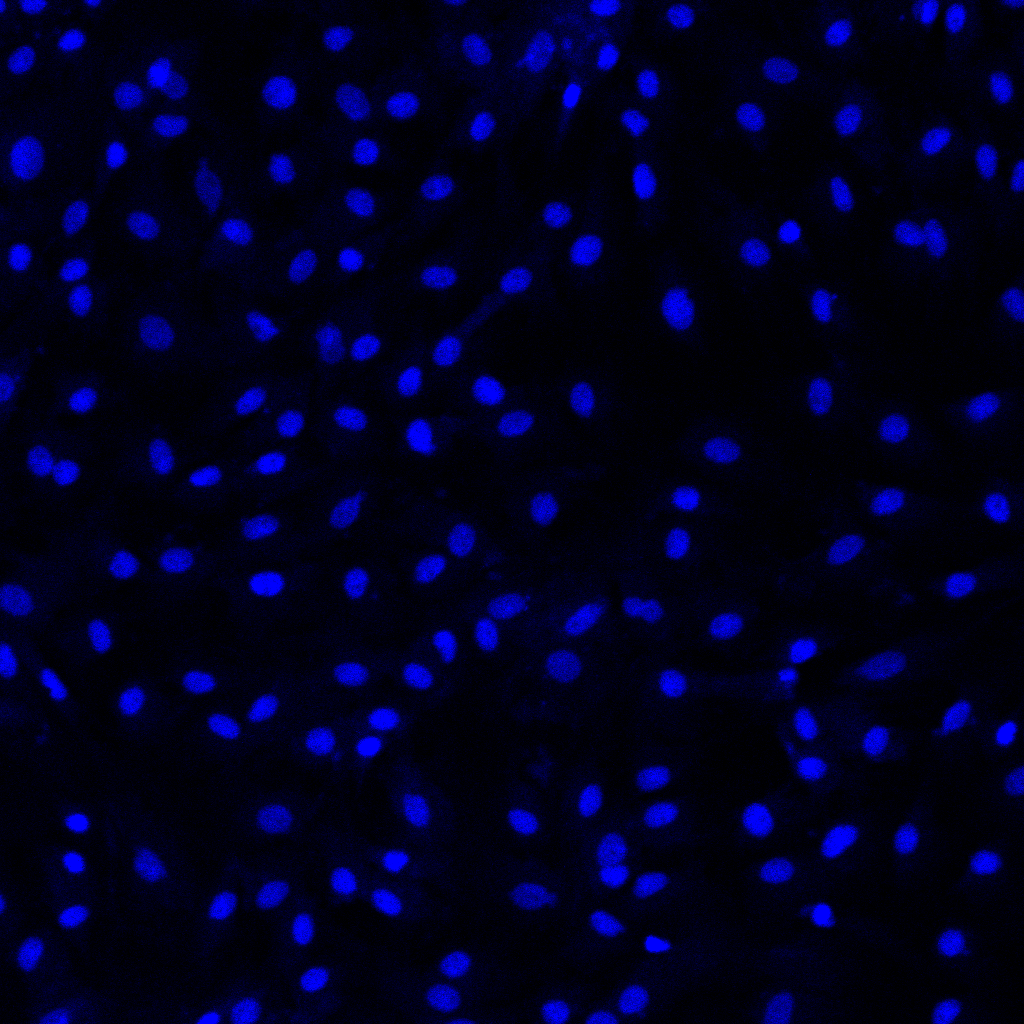

Supplement: Supplementary file 11 [file DataSheet7.ZIP › figure 5 H/circ-ATXN2-dapi.tif]

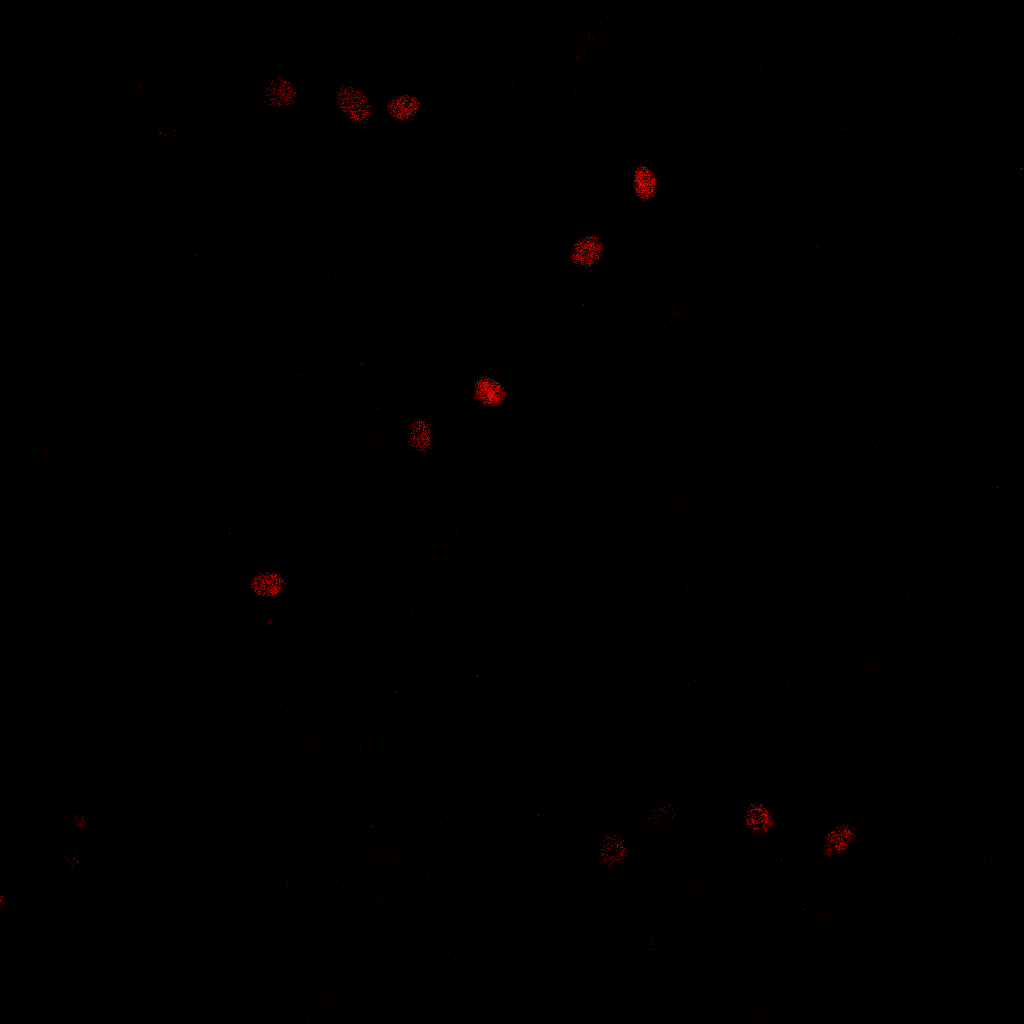

Supplement: Supplementary file 11 [file DataSheet7.ZIP › figure 5 H/circ-ATXN2-EDU.tif]

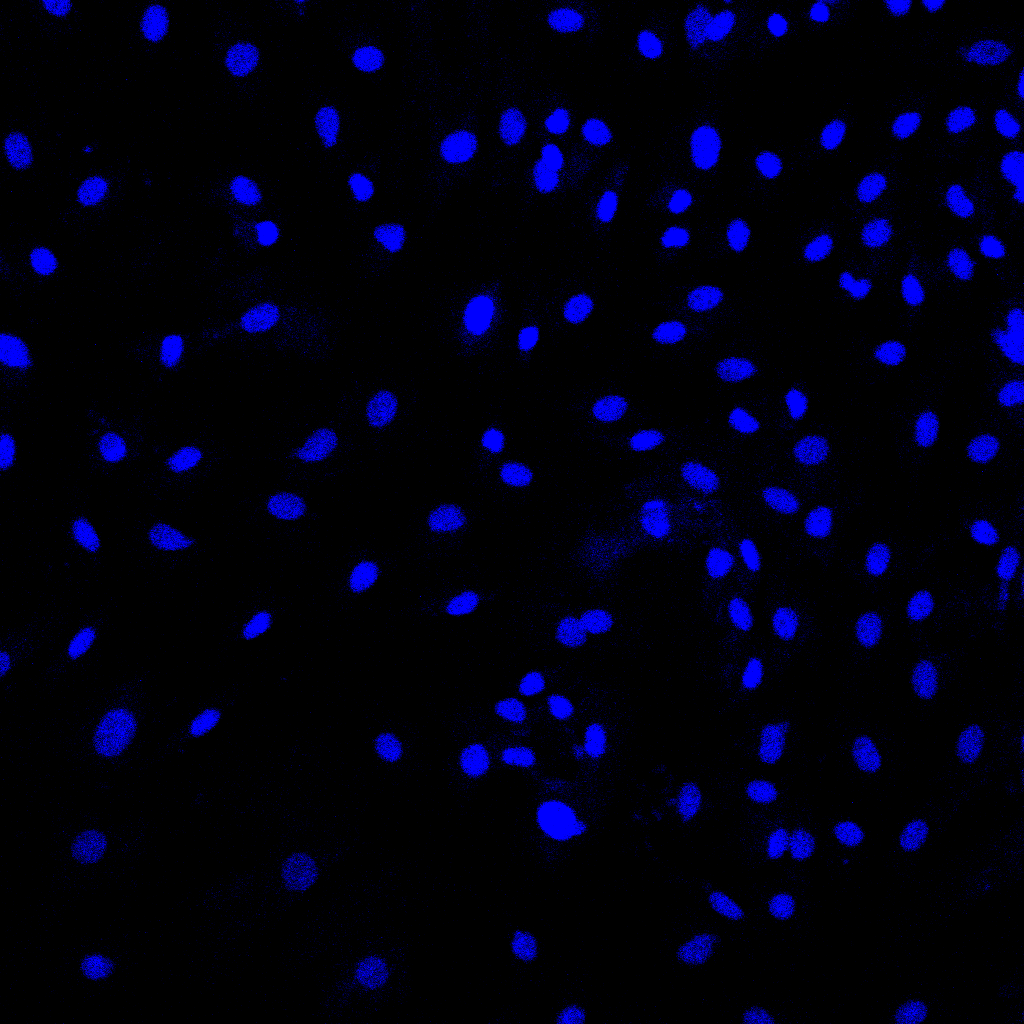

Supplement: Supplementary file 11 [file DataSheet7.ZIP › figure 5 H/VECTOR-dapi.tif]

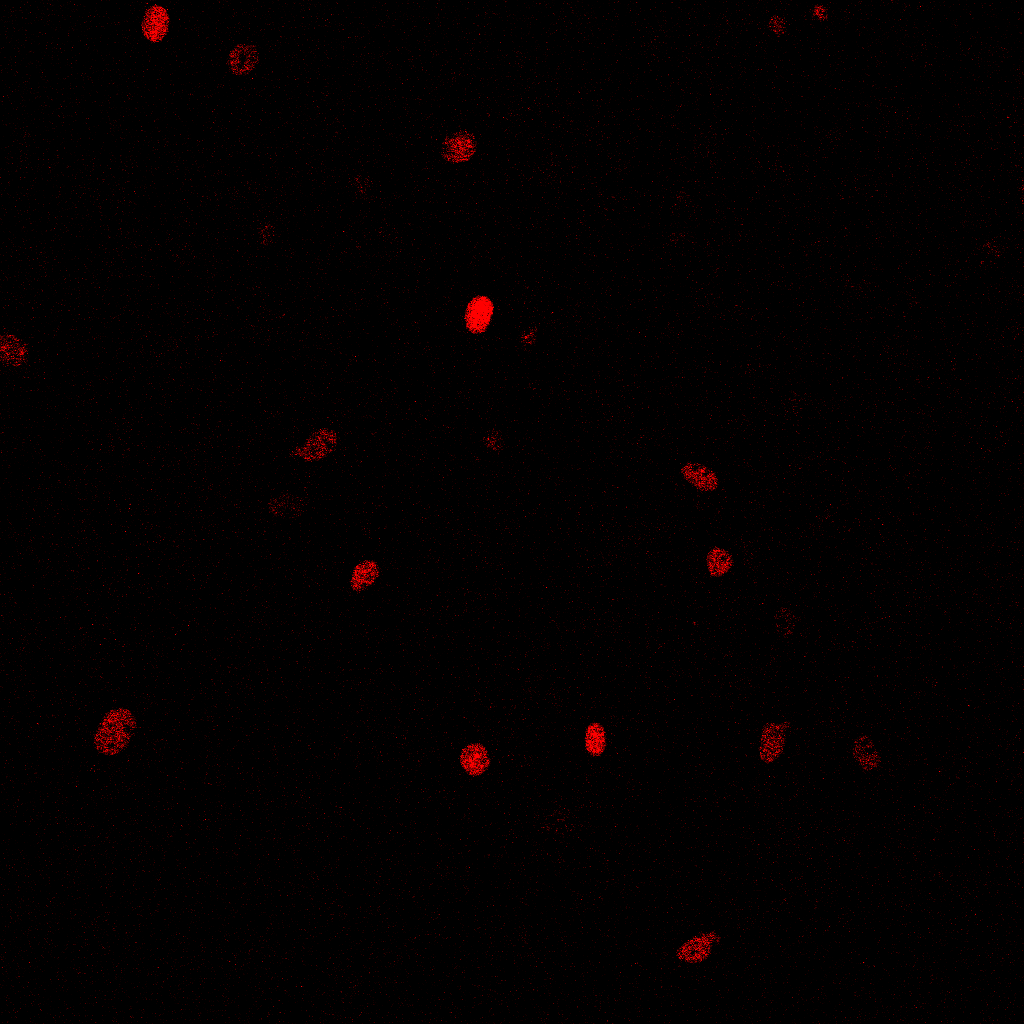

Supplement: Supplementary file 11 [file DataSheet7.ZIP › figure 5 H/VECTOR-EDU.tif]

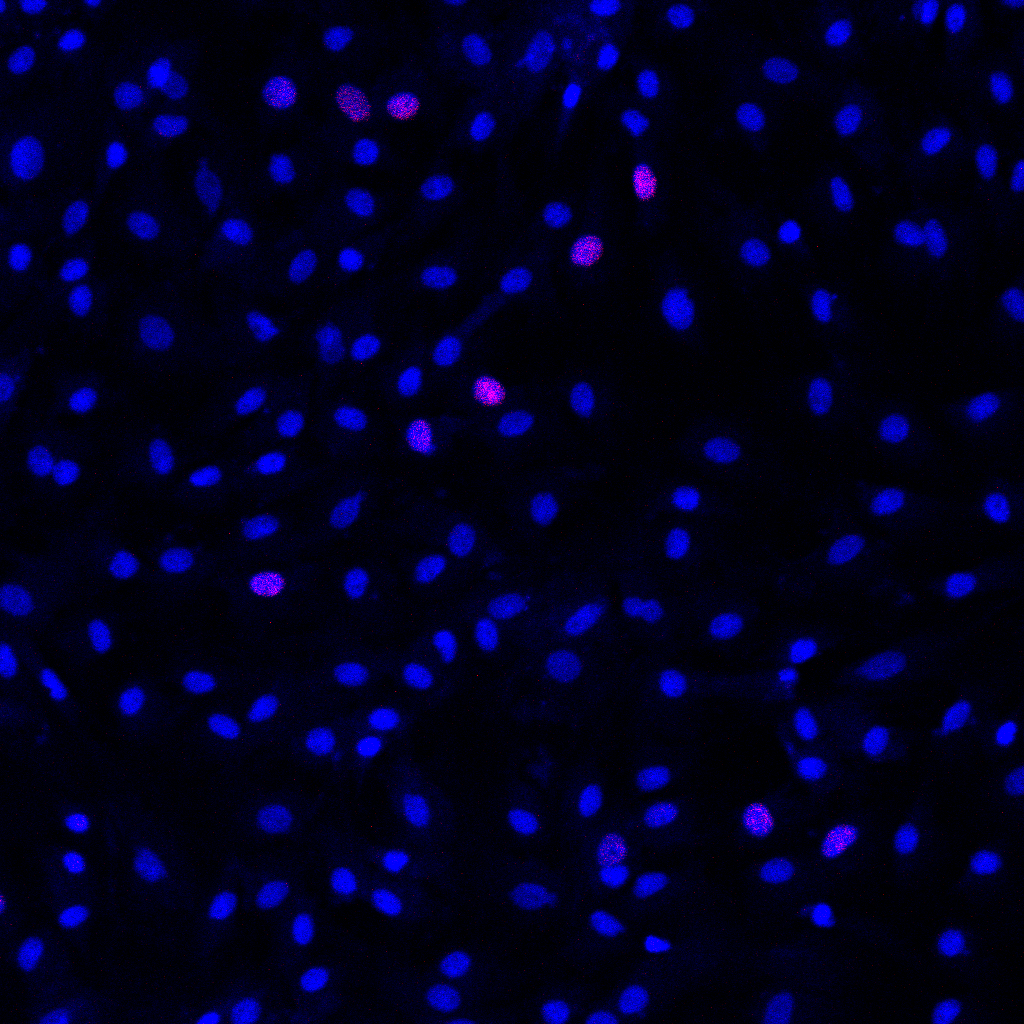

Supplement: Supplementary file 11 [file DataSheet7.ZIP › figure 5 H/circ-ATXN2-Composite.tif]
